# Tumor-derived PRMT1 suppresses macrophage antitumor activity by inhibiting cGAS/STING signaling in gastric cancer cells

**DOI:** 10.1038/s41419-025-07960-y

**Published:** 2025-08-26

**Authors:** Hui Wang, He Nie, Xiaoyi Zhao, Ke Chen, Chen Peng, Xuemei Zhang, Huiweng Guo, Yijia Chen, Xiaojing Yang, Dong Wang, Yongbing Yang, Qibin He, Qiang Zhan

**Affiliations:** 1 Department of Gastroenterology, The Affiliated Jiangning Hospital of Nanjing Medical University, Nanjing, 211100 Jiangsu China; 2 Departments of Gastroenterology, The Affiliated Wuxi People’s Hospital of Nanjing Medical University, Wuxi People’s Hospital, Wuxi Medical Center, Nanjing Medical University, Wuxi, Jiangsu 214023 P. R. China; 3 Jiangsu Branch of the National Clinical Research Center for Digestive Diseases, Wuxi, Jiangsu 214023 China; 4 Interdisciplinary Eye Research Institute (EYE-X Institute), Bengbu Medical University, Bengbu, Anhui China; 5 Anhui Provincial Key Laboratory of Immunology in Chronic Diseases, Bengbu, Anhui China

**Keywords:** Gastric cancer, Cancer microenvironment

## Abstract

Gastric cancer (GC) is a common and aggressive malignancy worldwide. Increasing evidence has shown that epigenetic changes are closely related to the development of cancer and tumor-associated macrophages. Here, we report that PRMT1 is a key immunosuppressive factor in GC. PRMT1 is upregulated in GC and promotes tumor progression. PRMT1 knockdown in GC leads to the activation of the cGAS/STING pathway through the enhancement of dsDNA aggregation, which subsequently increases IFN-β secretion. Notably, after PRMT1 knockdown, M1-like tumor-associated macrophage (TAM) infiltration increased, whereas M2-like TAM infiltration decreased in vivo and in vitro. After the targeted inhibition of STING by siRNA or H151, the improvement in the progression of GC caused by PRMT1 knockdown decreased, and the changes in macrophage polarization were reversed. Furthermore, we found that PRMT1 knockdown in GC affects the STAT pathway in TAMs, inducing changes in their polarization and promoting GC apoptosis by enhancing IFN-β secretion through the cGAS/STING pathway. In summary, our findings revealed that PRMT1 knockdown inhibits the cGAS/STING pathway in GC, which produces type I IFNs to promote the polarization of M1-like macrophages in the tumor microenvironment.

## Introduction

Gastric cancer (GC) is an aggressive and recalcitrant malignant tumor with the fifth highest incidence and the second highest mortality rate worldwide, seriously endangering public health [1]. Although the standard treatment for GC has significantly improved, it is still limited to surgical resection, chemotherapy and radiotherapy [2]. The survival rate of patients with GC has not significantly improved, with the 5 year overall survival rate remaining <30% [3]. Over the past few years, immune checkpoint inhibitors have demonstrated promising advantages in the treatment of various cancers, although their effectiveness has been disappointing in GC patients [4]. Thus, finding an effective therapeutic target to increase the sensitivity of GC to immune checkpoint inhibitors is essential.

PRMT1, a main member of the protein arginine methyltransferases (PRMTs), plays a role in a variety of cellular processes, including transcriptional regulation, RNA processing, DNA damage repair, and signal transduction [5, 6]. Noteworthily, the overexpression of PRMT1 has been associated with the progression of various cancers through its transcriptional effects on carcinogenic factors [7, 8]. Previous studies have reported a strong correlation between PRMTs and the anticancer immune response, suggesting the key role of PRMTs in modulating the effectiveness of immunotherapy [9]. For example, alterations in PRMT1 function can modulate B-cell proliferation, differentiation, and survival in humoral immunity [10]. Moreover, PRMT1 is highly expressed in T cells and increases the expression of IL-10 and IL-6 to inhibit tumor growth in hepatocellular carcinoma [11]. Therefore, manipulating PRMT1 may be a viable strategy for modulating the tumor microenvironment. However, the specific role of PRMT1 in the GC tumor immune microenvironment (TME) has not been reported.

The TME is important for the development and progression of GC, consisting of a complex community composed of cancer cells, immune cells and supporting cells [12]. Notably, macrophages are the most abundant infiltrating immune cells in the TME of patients with GC, and the M1 phenotype acts as a proinflammatory agent, whereas the M2 phenotype promotes tumor progression [13]. Owing to their phenotypes and functions, targeting the TME, particularly by polarizing macrophages into a proinflammatory phenotype, may become a new strategy for future tumor immunotherapy in patients with GC. However, evidence for the interaction of PRMT1 with macrophages is still scarce. Understanding the relationship between PRMT1 and macrophages in GC is critical for the development of more effective treatment strategies.

Given the potential role of PRMT1 in antitumor immunity and the significant effect of the TME on GC progression, we established this study to explore the specific role of GC-derived PRMT1 and its downstream effects in tumor immune microenvironment. We identified PRMT1 in GC cells as a prognostic factor that not only drives tumor-associated macrophages (TAMs) toward tumor-promoting subtypes but also reduces apoptosis in GC cells by suppressing the cGAS-STING pathway. Together, our work provides evidence for the role of PRMT1 in tumor immunotherapy and reveals a novel function of PRMT1 in macrophage polarization in GC.

## Methods

### Source of clinical samples

Two independent tissue groups of GC patients were obtained from Wuxi People’s Hospital affiliated to Nanjing Medical University (Cohort 1: 53 pairs of GC and adjacent normal tissue samples from 2012.01 to 2012.12, followed for 5 years; Cohort 2: 4 pairs of GC surgical specimens from 2022.09 and 2023.01). Each specimen was pathologically examined and graded by two experienced pathologists. The study was reviewed and approved by the institutional ethics board of Wuxi People’s Hospital (KY22097), and followed the ethical principles of the Declaration of Helsinki 1964.

### Cell lines

HGC27, AGS, L929 and MFC cells were obtained from American Type Culture Collection. All cells were cultured at 37 °C under 5% CO2 in either DMEM, F12K or RPMI-1640 supplemented with 10% FBS, 100 U/ml penicillin and 100 μg/ml streptomycin.

### Animals

All animals received humane care according to the criteria outlined in the ‘Guide for the Care and Use of Laboratory Animals’ (8th Edition), and the animal experiments protocols were approved by the Institutional Animal Care and Use Committee of Wuxi People’s Hospital affiliated to Nanjing Medical University, Jiangsu, China(DL2023011). C57BL/6 mice and BALB/c-nu mice were purchased from Charles River Company. For In vivo tumor growth experiments, male mice, aged 4–10 weeks were housed in cages of 6 animals per cage during experimentation and under PC2 specific-pathogen free conditions. Animals of the same sex were randomly assigned to experimental groups.

### Cell culture and cell lines

Mouse cell lines: MFC was cultured in RPMI 1640 media (Gibco) supplemented with 10% (v/v) FBS and 1% (v/v) penicillin/streptomycin at 37 °C in 10% CO_2_ incubator. L929 was cultured in DMEM media (Gibco) supplemented with 10% FBS, 1% (v/v) penicillin/streptomycin at 37 °C in 10% CO_2_ incubator.

Human GC cell lines: AGS and HGC27 were cultured in DMEM or F12K media (Gibco) supplemented with 10% (v/v) FBS and 1% (v/v) penicillin/streptomycin and at 37 °C in 10% CO2 incubator.

### Sequences, plasmids and transfection

#### Lentiviruses

Polybrene was used to transfect lentiviruses (Hanheng, China) into GC cells and cultured for 72 h. And then these cells were selected with 2 μg/ml purinomycin to produce stable cell lines.

#### Plasmid

Overexpression and enzyme activity mutant plasmids (Hanheng, China) were transfected into cells using Lipofectamine 3000 (Invitrogen), and the cell lines were collected after 72 h of culture for subsequent experiments.

#### SiRNA

SiRNA (Ruibo, China) were incubated with targeting cells supplemented with RNAimax (Invitrogen) for 72 h. The sequences of siRNAs, shRNA and plasmid are listed in Table S1.

### Western blotting

Western blotting was performed as previously described. All primary antibodies were diluted at 1:1000 in TBST buffer containing 5% skim milk. Primary antibodies, including: PRMT1 (CST, Cat#2449), HSP70 (CST, Cat#4872), phospho-TBK1 (CST, Cat#5483), phospho-IRF3 (CST, Cat#4947), cleaved caspase3 (CST, Cat#9661), CD206 (CST, Cat#24595), CD86 (CST, Cat#91882), phospho-Stat1 (CST, Cat#9167), phospho-Stat2 (CST, Cat#88410), H4R3me2a (Invitrogen, Cat#PA5-102612), Histone H4(CST, Cat#2592) and actin (CST, Cat#4970).

### Cell proliferation experiment

For Cell Counting Kit 8 (CCK-8) assays, transfected cells were cultured in 96-well plates (3000 cells/Wells). After 0, 24, 48, 72 and 96 h, respectively, a solution of 10 μL CCK-8 was added to each well and the plate was incubated at 37 °C for 2 h. Finally, the 450 nm absorbance (450 OD value) of each well were evaluated using a microplate reader.

For Apoptosis assays, 50,000 cells/well of cells were cultured in 6-well plates and then was performed followed by protocol for Apoptosis Assay by flow cytometry Using Annexin V/PI Staining method. Proportion of apoptosis cells was then racked.

### ELISA

The concentrations of IFN-β in culture supernatants were measured by ELISA Kits (MULTI SCIENCES, Cat#EK2236/EK1236).

### Immunofluorescence staining and microscopy

Cells were seeded on a cover slide in 24-well plate and cultured overnight. After immobilized and permeated with TPBS, Cells were blocked with 2% BSA. Incubated overnight with the primary antibody at 4 °C, cells were washed three times in PBS and incubated with the secondary antibody at room temperature for 1 h. After staining with DAPI (Abcam, Cat#ab104139), the slides were imaged with microscopy (Leica Microsystems).

### Comet assay

Samples were prepared in strict accordance with the guidelines (Beyotime, Cat#C2041M). The subsequent processing involved a series of sequential steps, including sample preparation, cell lysis, DNA denaturation, electrophoresis, neutralization, and staining. Finally, images were captured with a confocal microscope and subsequently analyzed using Comet Assay Software Project.

### Antibodies and flow cytometry (FACS)

The following antibodies were used for flow cytometry: CD206 (eBioscience, Cat#12-2061-80), CD11b (Biolegend, Cat#W6/32), F4/80 (eBioscience, Cat#17-4801-82) and CD86 (Biolegend, Cat#W6/32). All FACS were analyzed using BD FACS Symphony A3 and FlowJo v10 software. FACS staining was performed in FACS buffer.

### Quantitative polymerase chain reaction (qRT-PCR)

To obtain total RNA, cells (>5 × 10^5^) were collected and purified using the RNeasy Mini Kit (Qiagen, Cat#74106) in accordance with the manufacturer’s instructions. qRT-PCR was conducted using the Power SYBR green PCR Master Mix (Applied Biosystem, Cat#4368708) and analyzed by delta delta Ct method. The qRT-PCR primer sequences were listed in Table S2.

### RNA sequencing

Total triplicated RNA was extracted from tissues transfected with shPRMT1 and negative ones. Followed by the manufacturer’s recommendation, RNA libraries were generated using Illumina TruseqTM RNA based on the HiSeq platform. And corresponding, cluster analysis was generated in cBot cluster. Read counts were converted to log2-TPM, and statistically significant differentially expressed genes were obtained by filtering with *p* < 0.05. GSEA pre-sequencing was performed using genomic information from the Molecular Marker Database (MSigDB) and the Kyoto Encyclopedia of Genes and Genomes (KEGG).

### In vivo analysis of tumor growth

HGC27 and MFC cells were electroporated with shRNA targeting PRMT1 or plasmids encoding PRMT1 enzyme activity mutants. Subsequently, 1 × 10^6^ cells were subcutaneously injected into recipient C57BL/6 mice or BALB/c-nu mice, with 3–4 mice per group. On days 4, 8, and 12 post-inoculation, mice were intraperitoneally injected with H151 (750 nmol/mouse). On the day of inoculation and every 3 days thereafter, clodronate liposomes (10 μL/g) was intraperitoneally injected. After inoculation, MS023 (80 mg/kg) was intraperitoneally injected at a frequency of 3 days of injection followed by 4 days of discontinuation. The development of subcutaneous tumors was monitored every 3 days, and tumor size was quantified using the formula: tumor volume = (length × short diameter²) / 2. Approximately 1 month after tumor cell implantation, subcutaneous tumor tissues were harvested for subsequent experiments.

### Immunohistochemistry (IHC)

For IHC staining sections were incubated with a primary antibody coupled to a goat and rabbit secondary antibody conjugated with mountain root peroxidase. All sections were examined by two blinded experienced pathologists, who independently scored the staining of each of the indicators in the tissue. Rare inconsistencies in scores are resolved through re-examination of slides and consultation between pathologists. The primary antibodies were PRMT1 (CST, Cat#2449), phospho-TBK1 (CST, Cat#5483), phospho-IRF3 (CST, Cat#4947), cleaved caspase3 (CST, Cat#9661), CD206 (CST, Cat#24595) and CD86 (CST, Cat#91882).

Score rules: (1) For the evaluation of immune cell infiltration, the percentage of positive cells was calculated, and the averaged result was obtained from 6 randomly selected microscopic fields of each section. Representative images were taken under a microscope. (2) Other indicators were analyzed according to the staining intensity multiplied by the staining positive area. Score of staining positive area: 0 = 0%, 1 = 1–25%, 2 = 26–50%, 3 = 51–75%, and 4 = 76–100%. The staining intensity was 0 as no staining, 1 as weak positive, 2 as medium positive, and 3 as strong positive.

### Macrophage polarization assays

Macrophages of human origin: Peripheral blood samples were provided by healthy blood donors recruited at the Wuxi People’ s Hospital from July 2022 to October 2024. Briefly, peripheral blood mononuclear cells (PBMC) were isolated by Ficoll (HyClone, Cat#17-1440-03) medium density gradient centrifugation. The adherent cells of PBMC were mainly monocytes and dendritic cells, and the adherent time was 15 min.Then, the cells were washed twice with PBS (phosphate buffered saline), and the cells were re-suspended at 2 × 10^6^ cells/mL with complete medium (DMEM, 10%FBS, 1% double antibody, 20 ng/ml M-CSF) and placed on a 12-well plate. The fluid was changed every 3 days, and the follow-up experiment was conducted after 7 days.

Mouse derived macrophages: from wild-type C57BL/6 mice, the bone marrow cavity was rinsed with complete medium inside the tibia of the mouse. The medium with bone marrow cells was collected into a centrifuge tube and centrifuged at 1500 rpm for 5 min to remove the supernatant. The sediment was re-suspended in complete medium (DMEM, 10% FBS, 1% penicillin and streptomycin, 10% L929) and cultured in a six-well plate (1 × 10^6^ cells/well) for 2 days. Subsequently, the supernatant was collected and centrifuged, and then re-suspended with complete medium and planted back into the original bottle for further culture. Then the cell fluid was changed every three days normally, and the follow-up experiment was conducted after 7 days of culture.

These macrophages (1 × 10^6^ cells/well), seeded in the bottom layer of a 6-well transwell culture plate, were co-cultured with GC cells in the upper chamber for 48 h. Then macrophages in the bottom chamber were harvested.

### Statistical analysis

All data were collected from >3 independent experiments. Unless otherwise noted, results were expressed as mean ± standard deviation and analyzed using GraphPad Prism9 software (GraphPad Software, San Diego, CA). The significance of differences was derived by using the two-tailed *T*-test, Kaplan-Meier survival curve, logarithmic rank test and Chi-square test. *P* < 0.05 was deemed statistically significant.

## Results

### PRMT1 overexpression was linked to poor prognosis in GC patients and promoted the progression of GC cells in vitro and in vivo

**Fig. 1:**
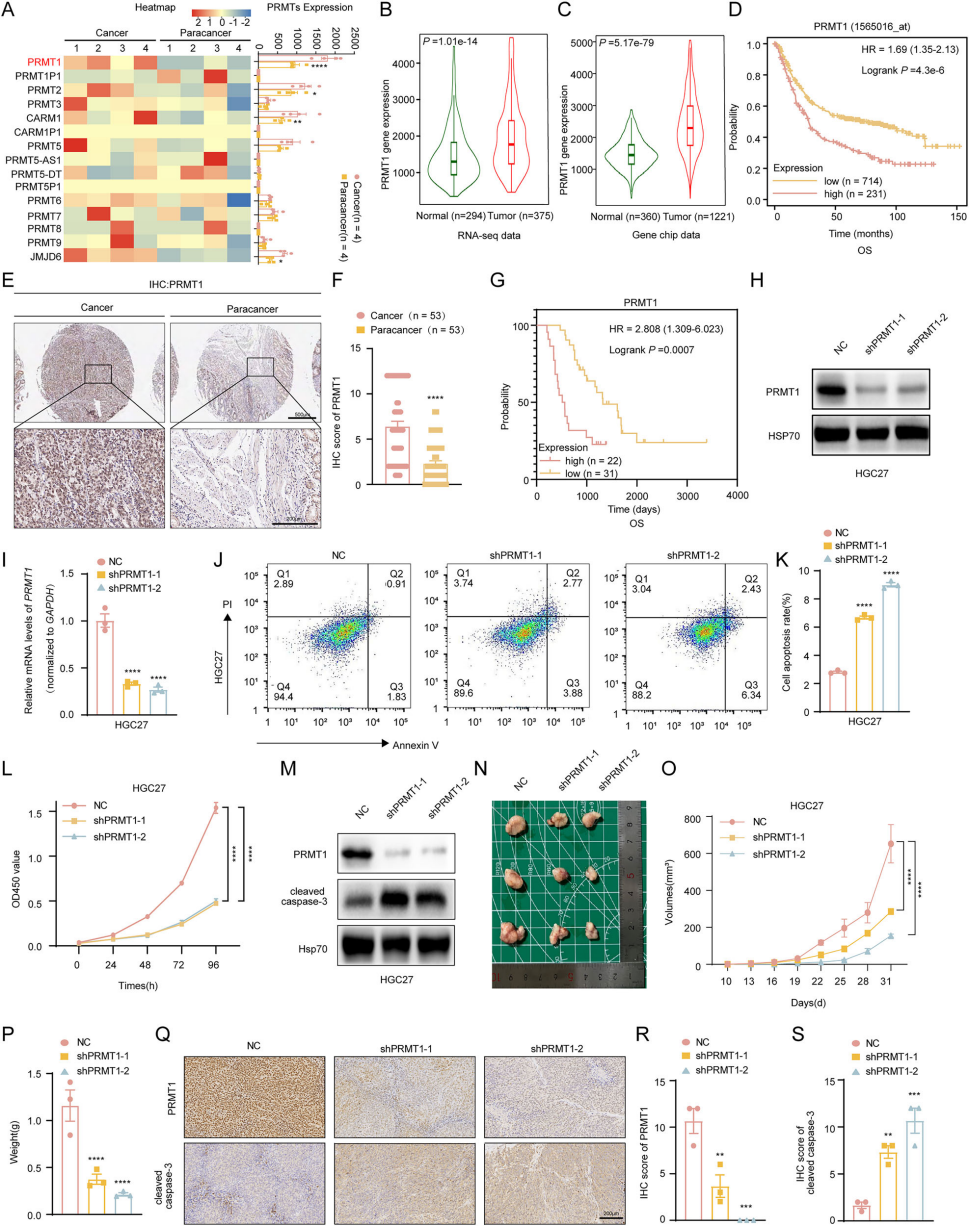
Clinical expression, prognostic value and cancer promoting functions of PRMT1 in GC. **A** Heatmap of PRMTs family (left) and its expressions (right) from RNA-seq in 4 pairs of cancer and paracancer tissue in GC patients. **B**-**C** Public database-based analysis depicted PRMT1 mRNA expression in cancer and normal tissues of GC patients through RNA-seq data and Gene Chip data. **D** Kaplan-Meier curves of overall survival in 945 patients with GC, stratified by PRMT1 expression (Log-rank test, *P* = 0.00043) (https://kmplot.com/analysis/index.php?p=service&cancer=gastric). **E**, **F** Representative microphotographs of IHC staining (E) and IHC scores (**F**) for PRMT1 in a tissue chip with GC and paired para-tumor normal tissues from the clinic. Magnification: 10 × and 400 ×. **G** Kaplan-Meier curves of overall survival in 53 patients with GC in the tissue chip, stratified by PRMT1 expression (Log-rank test, *P* = 0.0007). **H**-**I** PRMT1 expression levels of HGC27 after lentivirus interference detected by Western blots (**H**) and qRT-PCR (I). **J**-**K** Flow cytometry analysis of the apoptotic cells after depletion of PRMT1 in HGC27 cells using the Annexin V-APC/PI staining assay (J). Positive Annexin V cells were displayed as a histogram (K). **L** The proliferation rates of HGC27 cells upon depletion of PRMT1 were determined by CCK8 assay. (**M**) Western blots detected protein levels of PRMT1 and cleaved caspase-3 in HGC27 after down-expressing PRMT1. **N** Resected tumors formed in nude mice injected with the indicated cells from each group (*n* = 3) were photographed. **O**-**P** The volumes and weights of resected tumors from each mouse were recorded. **Q**-**S** Representative images of IHC staining (**Q**) and IHC scores (**R**-**S**) of PRMT1 and cleaved caspase-3 in resected tumors from nude mice. *P < 0.05, **P < 0.01, ***P < 0.001, ****P < 0.0001.

To investigate genes that are relatively highly expressed in GC tissues, we extracted RNA from 4 sets of fresh cancerous and paracancerous tissues from patients with GC and performed RNA-seq analysis, revealing that PRMT1 is relatively highly expressed within the PRMT family (Fig. 1A and Supplementary Fig. 1A). To further verify the expression of PRMT1 in GC, we analyzed its mRNA expression in a public database of GC patients and found that PRMT1 was highly expressed in GC tissues compared with normal tissues; its expression was also significantly negatively correlated with the prognosis of GC patients (Fig. 1B–D and Supplementary Fig. 1B, C). Additionally, the results of IHC in 53 collected GC pathological samples revealed that the protein expression level of PRMT1 in GC tissues was significantly greater than that in adjacent tissues and that PRMT1 was primarily localized in the nucleus (Fig. 1E, F). As shown in Fig. 1G, the overall survival rate of the group with high PRMT1 expression was significantly lower than that of the group with low PRMT1 expression. These data suggest that elevated expression of PRMT1 has pathological significance in the development of GC.

**Fig. 2:**
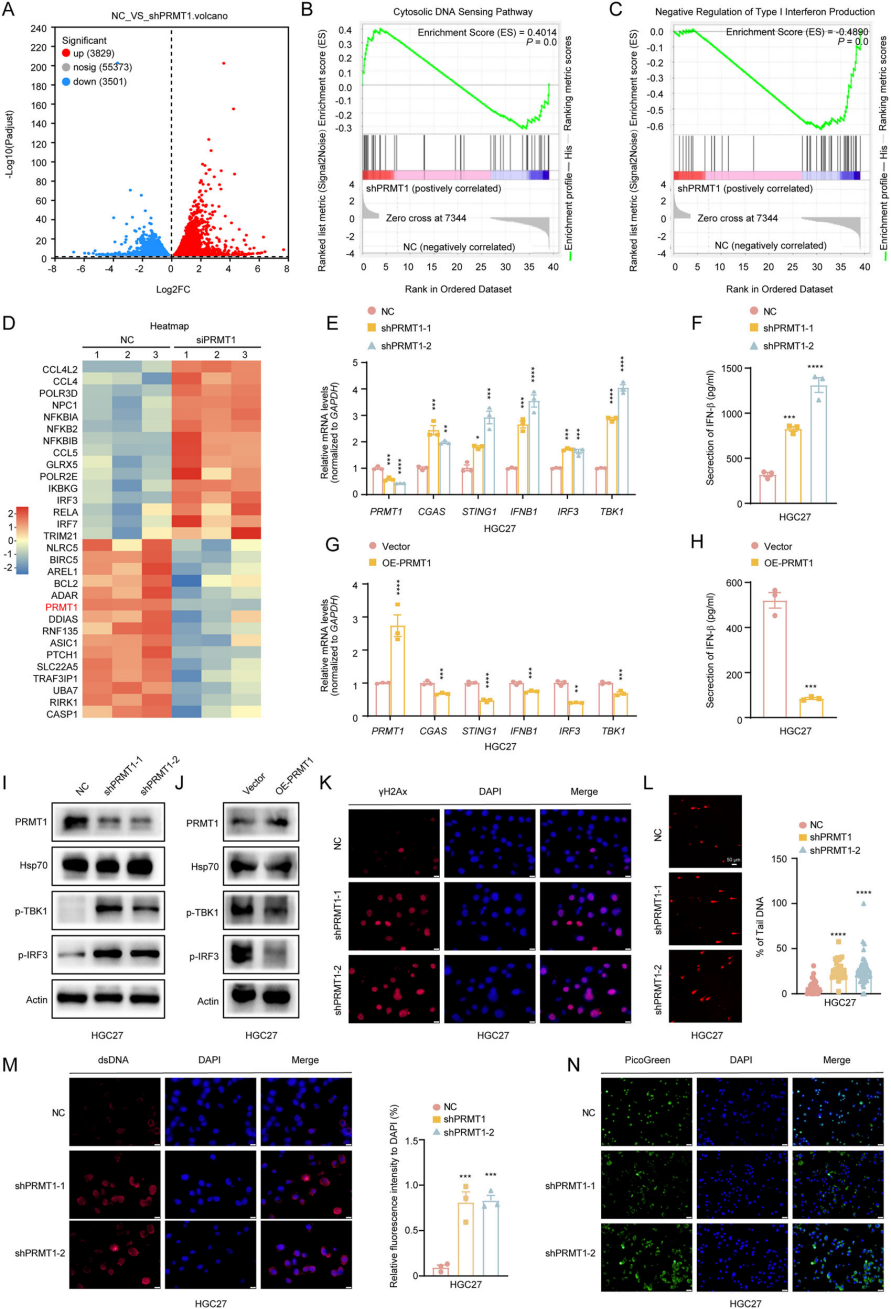
PRMT1 knockdown triggered an antitumor response via the activation of cGAS/STING signaling. **A** The volcano chart of HGC27 cells after PRMT1 knockdown (blue: 3501 down-regulated genes; red: 3829 upregulated genes). **B**, **C** GSEA plots for cytosolic DNA-sensing pathway (**B**) and type I IFN production (**C**) related genes in HGC27 cells following knocking down PRMT1. **D** Heatmap of the top 15 upregulated and down-regulated genes in HGC27 cells after PRMT1 knockdown. The data presented was normalized by log2. **E** The mRNA expression of CGAS, STING1, IFNB1, TBK1, and IRF3 in HGC27 cells after PRMT1 knockdown. **F** The expression levels of IFN-β in the secretion of HGC27 cells after PRMT1 knockdown were detected by ELISA. **G** The mRNA expression of CGAS, STING1, IFNB1, TBK1, and IRF3 in HGC27 cells after PRMT1 overexpression. **H** The expression levels of IFN-β in the secretion of HGC27 cells after PRMT1 overexpressed were detected by ELISA. **I**, **J** Protein levels of PRMT1, p-TBK1, and p-IRF3 in HGC27 cells after PRMT1 was downregulated (**I**) or up-regulated (**J**) were detected by Western blot. **K** For immunofluorescent staining, HGC27 cells were stained with anti-γH2AX antibody (red) and counter-stained with DAPI (blue) after PRMT1 was downregulated. Magnification: 200 ×. **L** Representative images of the comet assay of HGC27 cells after PRMT1 was downregulated. Magnification: 100 ×. **M** For immunofluorescent staining, HGC27 cells were stained with anti-dsDNA antibody (red) and counter-stained with DAPI (blue) after PRMT1 was downregulated (left). Relative fluorescence intensity of dsDNA to DAPI was displayed as a histogram (right). Magnification: 200 ×. **N** Representative images of HGC27 cells using PicoGreen (green) and DAPI (blue). Magnification: 200 ×. *P < 0.05, **P < 0.01, ***P < 0.001, ****P < 0.0001.

To further investigate the role of PRMT1 in gastric cancer (GC), we successfully established stable GC cell lines (HCG27, AGS, and MFC) with PRMT1 knockdown, overexpression, and enzyme activity mutations using lentiviral vectors to transduce short hairpin RNA (shRNA) and PRMT1 enzyme mutant plasmids (Fig. 1H, I, Supplementary Fig. 1D, E, supplementary Fig. 2A–C, supplementary Fig. 2K–M and supplementary Fig. 2U). Using these cell lines, we sought to assess whether PRMT1 had an effect on GC cell proliferation. As shown in the figures, the apoptosis rate of the GC cells significantly increased after knocking down the expression of PRMT1 or mutating the enzyme activity of PRMT1, as determined via flow cytometry (Fig. 1J, K, supplementary Fig. 2D–G and supplementary Fig. 2V–W). The results of the CCK8 experiment revealed that the proliferation ability of GC cells was significantly inhibited after the knockdown or enzyme mutation of PRMT1 (Fig. 1L, supplementary Fig. 2H, I and Fig. 2X). The downregulation of PRMT1 subsequently activated the shear-induced activation of caspase3 (Fig. 1M and supplementary Fig. 2J). These results suggest that PRMT1 knockdown inhibits the proliferation of GC cells. Moreover, we also found that the overexpression of PRMT1 in GC cells significantly increased the proliferation of GC cells (supplementary Fig. 1F–I and supplementary Fig. 2N–T). Therefore, further studies on the role of PRMT1 in xenotransplantation mouse models are urgently needed.

Since results above showed that PRMT1 was upregulated in human GC tissues and affected cell proliferation, we decided to investigate whether PRMT1 knockdown could inhibit tumor growth in vivo. We injected HGC27 cells (control shRNA, shPRMT1-1, and shPRMT1-2) subcutaneously into nu/nu nude mice. As shown in Fig. 1N–P, the subcutaneous tumor tissue size and tumor weight significantly decreased after PRMT1 knockdown. Moreover, IHC staining revealed significantly increased cleaved caspase3 expression after PRMT1 knockdown in vivo (Fig. 1Q–S). Collectively, our results demonstrated that targeting PRMT1 significantly promoted the anticancer effect of GC both in vitro and in vivo.

### PRMT1 knockdown triggered an antitumor response via the activation of cGAS/STING signaling

To explore the underlying mechanism by which PRMT1 promotes tumorigenesis, we performed RNA-seq analysis on control and PRMT1 knockdown GC cells (Fig. 2A). The volcano map presents upregulated 3829 genes and 3501 downregulated genes in PRMT1-deficiency GC cells (Fig. 2A). Interestingly, GSEA and heatmap revealed that the cytosolic DNA-sensing pathway and type I IFN production were significantly upregulated after the deletion of PRMT1 (Fig. 2B–D). Since the type I IFN production signal and the cytosolic DNA-sensing pathway correspond to the cGAS/STING pathway [14], we speculated that PRMT1 negatively regulates the cGAS/STING signal of GC cells.

To test this hypothesis, we verified changes in the cGAS/STING signaling pathway in GCs after PRMT1 expression changed. First, the mRNA levels of *CGAS*, *STING1*, *IFNB1*, *TBK1* and *IRF3* were significantly increased after PRMT1 knockdown when compared with control group (Fig. 2E, supplementary Fig. 3B, D). Second, western blot results confirmed that the phosphorylation levels of TBK1 and IRF3 was elevated after PRMT1 knockdown (Fig. 2I and supplementary Fig. 3A). Third, IFN-β secretion increased in GC cells after PRMT1 knockdown, as confirmed via ELISA (Fig. 2F, supplementary Fig. 3C, E). Moreover, we also found that the overexpression of PRMT1 in GC cells significantly inhibited the cGAS/STING signaling pathway in GC cells (Fig. 2G, H, J, supplementary Fig. 3F–J). Thus, these results suggest that PRMT1 regulates the cGAS/STING signaling pathway in GC cells.

It has been reported that DNA damage can lead to increased accumulation of cytoplasmic dsDNA, which is then recognized by cGAS, activating the STING pathway [15]. Therefore, we investigated whether PRMT1 in GC cells could increase DNA damage, leading to the accumulation of dsDNA and then activating the cGAS/STING signaling. As expected, the DNA damage marker γH2AX was significantly increased in PRMT1-knockdown GC cells (Fig. 2K, supplementary Fig. 3K, N). The results of the comet assay showed that after knockdown of PRMT1, DNA damage in gastric cancer cells increased (Fig. 2L, supplementary Fig. 3P, Q). We also observed increased cytoplasmic dsDNA accumulation when PRMT1 was knocked down in GC cells (Fig. 2M, N, supplementary Fig. 3L, M, O). In summary, these data suggested that targeting PRMT1 activates the cGAS/STING signaling pathway through the accumulation of dsDNA in GC cells, leading to an increase in type I interferon secretion.

### PRMT1 knockdown/enzyme activity mutation leads to increased infiltration and polarization of M1-like TAMs in GC to improve antitumor ability

**Fig. 3:**
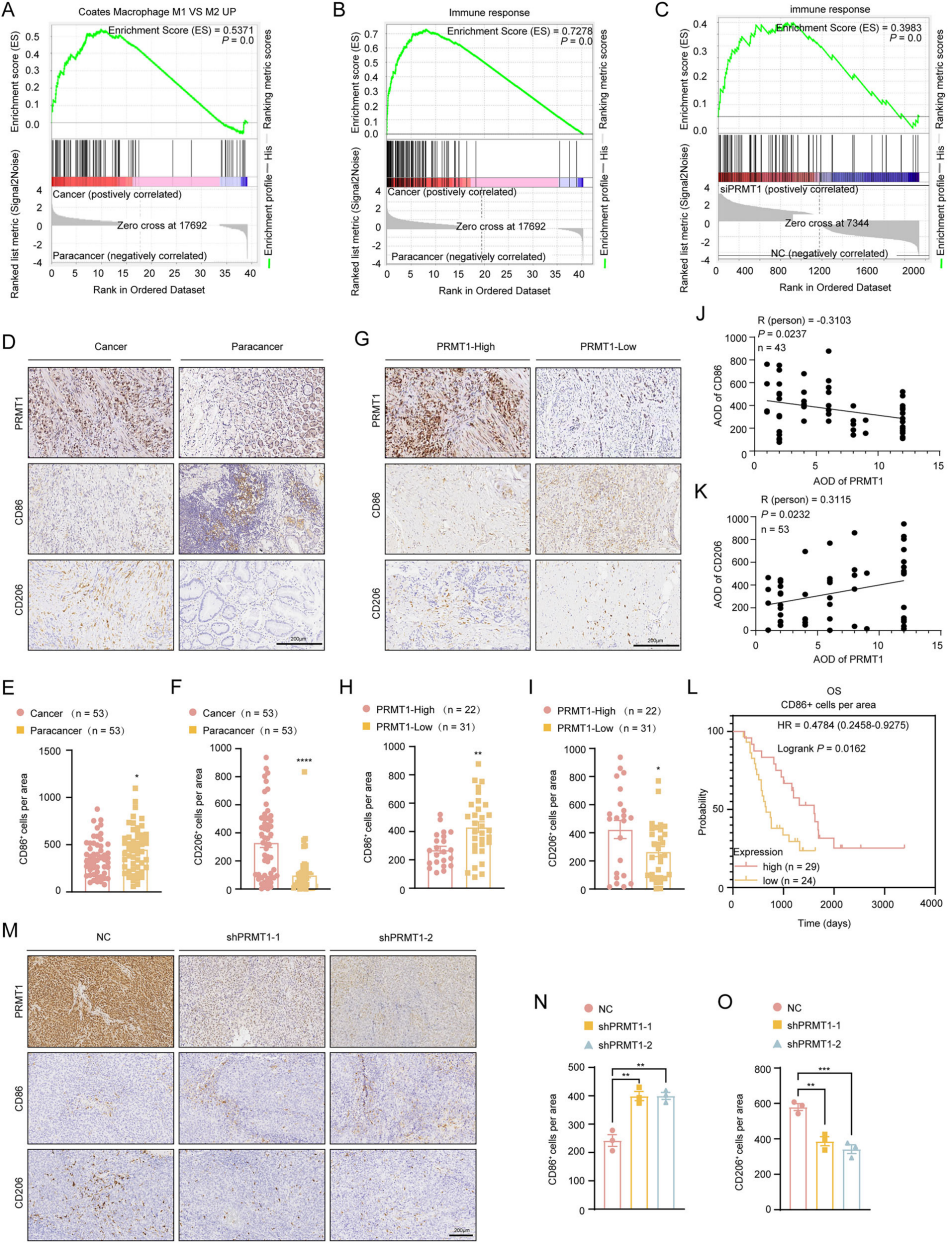
Clinical expression and prognostic value of M1 and M2 macrophages in GC, especially the relationship between PRMT1 expression and M1 and M2 macrophages in GC. **A**, **B** GSEA plots for macrophage M1 vs. M2-up (**A**) and immune response (**B**) related genes from RNA-seq in 4 pairs of cancer and paracancer tissue in GC patients. **C** GSEA plots for immune response related genes in HGC27 cells following knocking down PRMT1. **D**−**F** The changes in the infiltration of CD86^+^ and CD206^+^ macrophages in a tissue chip with GC and paired para-tumor normal tissues from the clinic. Representative images after staining (**D**) and statistical analysis of the CD86^+^ and CD206^+^ cells per area (**E**, **F**). Magnification: 400 ×. **G**−**I** The changes in the infiltration of CD86^+^ and CD206^+^ macrophages in PRMT1-High and PRMT1-Low groups in a tissue chip with GC and paired para-tumor normal tissues from the clinic. Representative images after staining (**G**) and statistical analysis of the CD86^+^ and CD206^+^ cells per area (**H**, **I**). Magnification: 400 ×. **J**, **K** Correlation analysis of PRMT1 and CD86^+^ or CD206^+^ cells per area based on IHC. (**L**) Kaplan-Meier curves of overall survival in 53 patients with GC in the tissue chip, stratified by CD86^+^ cells per area (Log-rank test, *P* = 0.0162). **M**−**O** Representative images of IHC staining (**M**) and positive cells per area (**N**-**O**) of CD86^+^ and CD206^+^ cells in resected tumors from nude mice in NC and shPRMT1 groups. Magnification: 400 ×. *P < 0.05, **P < 0.01, ***P < 0.001, ****P < 0.0001.

Since immune cells in TME regulates tumorigenesis in various tumor [16], we then explored the changes in the TME of patients with GC. Using the TIMER 2.0 website, we identified four risk factors for poor prognosis in 415 patients with GC: age, stage 3 GC, stage 4 GC and high macrophage infiltration. High macrophage infiltration is the most significant risk factor (Table 1). Further, we found the macrophage M1-vs.-M2-up, immune response and cytosolic DNA-sensing pathways were significantly upregulated in GC tumor tissue when compared with paracancerous tissues in the patient samples we sequenced before (Fig. 3A, B and Supplementary Fig. 4C). Therefore, these data suggest that immune cells or immune pathways may be involved in gastric antitumor activity.

**Table 1:** Cox regression analysis of prognostic factors for gastric cancer.

Description	coef	HR	95%CI_l	95%CI_u	*P*-value
**age**	0.037	1.038	1.016	1.06	0.001
**stage2**	0.739	2.095	0.919	4.775	0.079
**stage3**	1.085	2.961	1.376	6.372	0.006
**stage4**	1.444	4.237	1.479	12.138	0.007
**B cell**	4.682	107.989	0.835	13969.253	0.059
**CD8**^**+**^**Tcell**	−0.863	0.422	0.019	9.539	0.587
**CD4**^**+**^**Tcell**	−4.506	0.011	0.000	2.785	0.110
**Macrophage**	7.789	2413.552	55.410	105130.292	0.000
**Neutrophil**	−2.682	0.068	0.000	129.965	0.486
**Dendritic**	0.799	2.224	0.115	42.886	0.596

In accordance with previous studies, PRMTs regulate immune cells recruitment and activation in TME [17]. Next, we analyzed the results of the RNA-seq results in PRMT1-knockdown and control HGC27 cells. Compared with that in the control group, the immune response pathway was upregulated after PRMT1 was knocked down (Fig. 3C and Supplementary Fig. 4A, B). Additionally, we analyzed the correlation between PRMT1 expression and tumor-related immune cells via the TIMER website. The results suggested that PRMT1 in GC cells was positively correlated with macrophage and CD8 + T-cell infiltration and negatively correlated with CD4 + T-cell infiltration (Supplementary Fig. 4E, F). We then validated the results of the public database analysis in human gastric and para-cancerous tissues. IHC analysis of 53 GC pathological samples revealed that the number of tumor-infiltrating CD86^+^ M1 macrophages was significantly lower in GC tissues than in paracancerous tissues and that the number of CD206^+^ macrophages was significantly greater (Fig. 3D–F). Moreover, low expression of PRMT1 in GC tissues affects the infiltration of macrophages, with increasing the number of M1-like macrophages and decreasing the number of M2-like macrophages (Fig. 3G–K). As shown in Fig. 3L, the overall survival rate of the group with high numbers of tumor-infiltrating CD86^+^ M1 macrophages was significantly higher. However, the presence of tumor-infiltrating CD206^+^ M2 macrophages was not associated with the prognosis of GC patients (Supplementary Fig. 4D). Using subcutaneous tumor tissues in Fig. 1O, tumor-infiltrating CD86^+^ M1 macrophages was significantly higher in PRMT1 knockdown groups than control ones, as tumor-infiltrating CD206^+^ M2 macrophages significantly lower (Fig. 3M–O). These data clearly revealed that macrophages are essential for PRMT1 deficiency-mediated tumor suppression.

**Fig. 4:**
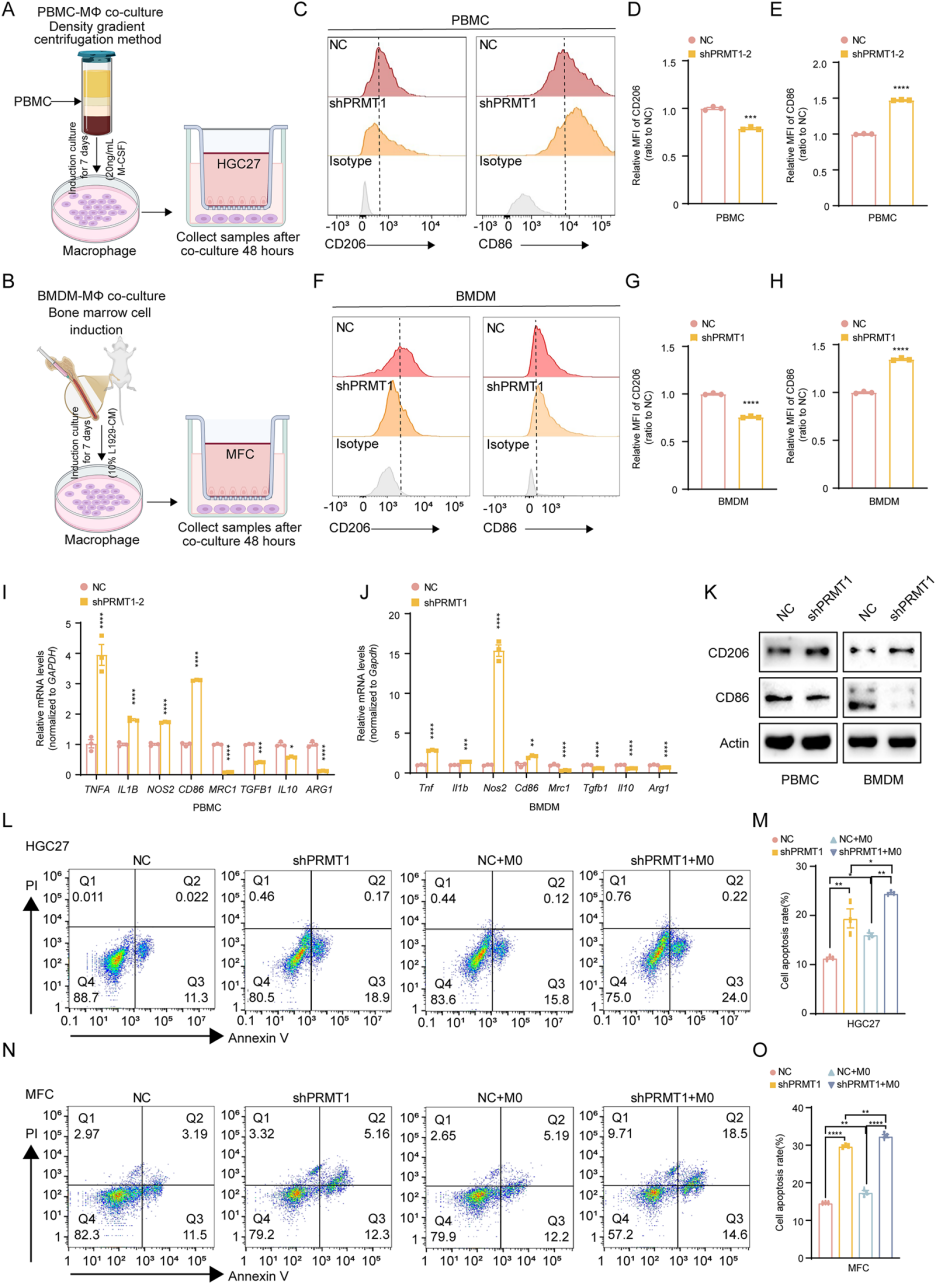
PRMT1 knockdown leads to increased infiltration and polarization of M1-like TAMs in GC to improve antitumor ability. **A**, **B** Schematic of the coculture of gastric cancer cells with PBMCs (**A**) or BMDMs (**B**) in the chamber system. **C**–**E** PBMCs from the coculture system with HGC27 cells, depleted or not depleted of PRMT1, and analyzed by flow cytometry with anti-CD86 and anti-CD206 antibodies (**C**). The ratio of MFI of CD86^+^ cells (**D**) and CD206^+^ cells (**E**) is shown. MFI mean fluorescence intensity. **F**−**H** BMDMs from the coculture system with MFC cells, depleted or not depleted of PRMT1, and analyzed by flow cytometry with anti-CD86 and anti-CD206 antibodies (**F**). The ratio of MFI of CD86^+^ cells (**G**) and CD206^+^ cells (**H**) is shown. **I**, **J** mRNA levels of M1 and M2 macrophage markers in PBMCs (**I**) and BMDMs (**J**) from the coculture system with GC cells after PRMT1 knockdown were detected by qRT-PCR. **K** Protein levels of CD86 and CD206 in PBMCs (left) and BMDMs (right) from the coculture system with GC cells after PRMT1 knockdown were detected by Western blot. **L**, **M** Flow cytometry analysis of the apoptotic HGC27 cells after depletion of PRMT1 from the coculture system with PBMCs using the Annexin V-APC/PI staining assay (**L**). Positive Annexin V cells were displayed as a histogram (**M**). **N**, **O** Flow cytometry analysis of the apoptotic MFC cells after depletion of PRMT1 from the coculture system with BMDMs using the Annexin V-APC/PI staining assay (**N**). Positive Annexin V cells were displayed as a histogram (**O**). *P < 0.05, **P < 0.01, ***P < 0.001, ****P < 0.0001.

Additionally, we conducted an in vitro coculture experiment to examine the effect PRMT1 expression levels in GC cells on the polarization of tumor-infiltrating macrophages (Fig. 4A, B). Flow cytometry revealed that the percentage of CD86^+^ macrophages was greater and that the percentage of CD206^+^ macrophages was lower in the shPRMT1 group than in the control group (Fig. 4C–H). Furthermore, western blot and qRT-PCR analyses revealed that the protein and mRNA levels of M2 macrophage markers were significantly reduced after PRMT1 knockdown in GC cells, whereas M1 macrophage markers were significantly increased (Fig. 4I–K). These results demonstrate that decreased PRMT1 expression in GC cells induces changes in macrophage polarization. Moreover, we detected flow cytometry-mediated apoptosis in GC cells cocultured with macrophages. After coculture with macrophages, tumor cell apoptosis increased, especially after PRMT1 knockdown in GC (Fig. 4L–O). Furthermore, we generated PRMT1 enzyme activity mutants in MFC cells and co-cultured with macrophages. Strikingly, PRMT1 enzyme activity mutation recapitulated the phenotypic changes observed in PRMT1-knockdown cells. Specifically, mutant PRMT1 significantly reduced CD206 expression while upregulating CD86 in macrophages (Supplementary Fig. 5A, B). In vivo experiments further demonstrated that PRMT1 enzyme activity mutation potently suppressed subcutaneous tumor growth (Supplementary Fig. 5C–E), concomitantly inhibiting M2 polarization and promoting M1 polarization of intratumoral macrophages (Supplementary Fig. 5F, G). These phenomena suggested that PRMT1 functional deficiency might drive the polarization of macrophages toward the M1-like TME in the GC microenvironment.

### PRMT1 knockdown-induced antitumor properties and polarization of M1-like macrophages through STAT signaling were abolished after inhibition of cGAS/STING signaling

As showed in Fig. 2, PRMT1 deficiency activates the cGAS/STING signaling and promotes type I interferon secretion, M1-like macrophages in the TME can be directly recruited by IFN-β [18]. Therefore, following PRMT1 knockdown in GC cells, the observed changes in M1 and M2 macrophages within tumors may be attributed to the production of IFN-β downstream of the cGAS-STING pathway in GC cells. To test this hypothesis, we inhibited the cGAS-STING pathway by silencing STING expression in PRMT1-deficient GC cells.

**Fig. 5:**
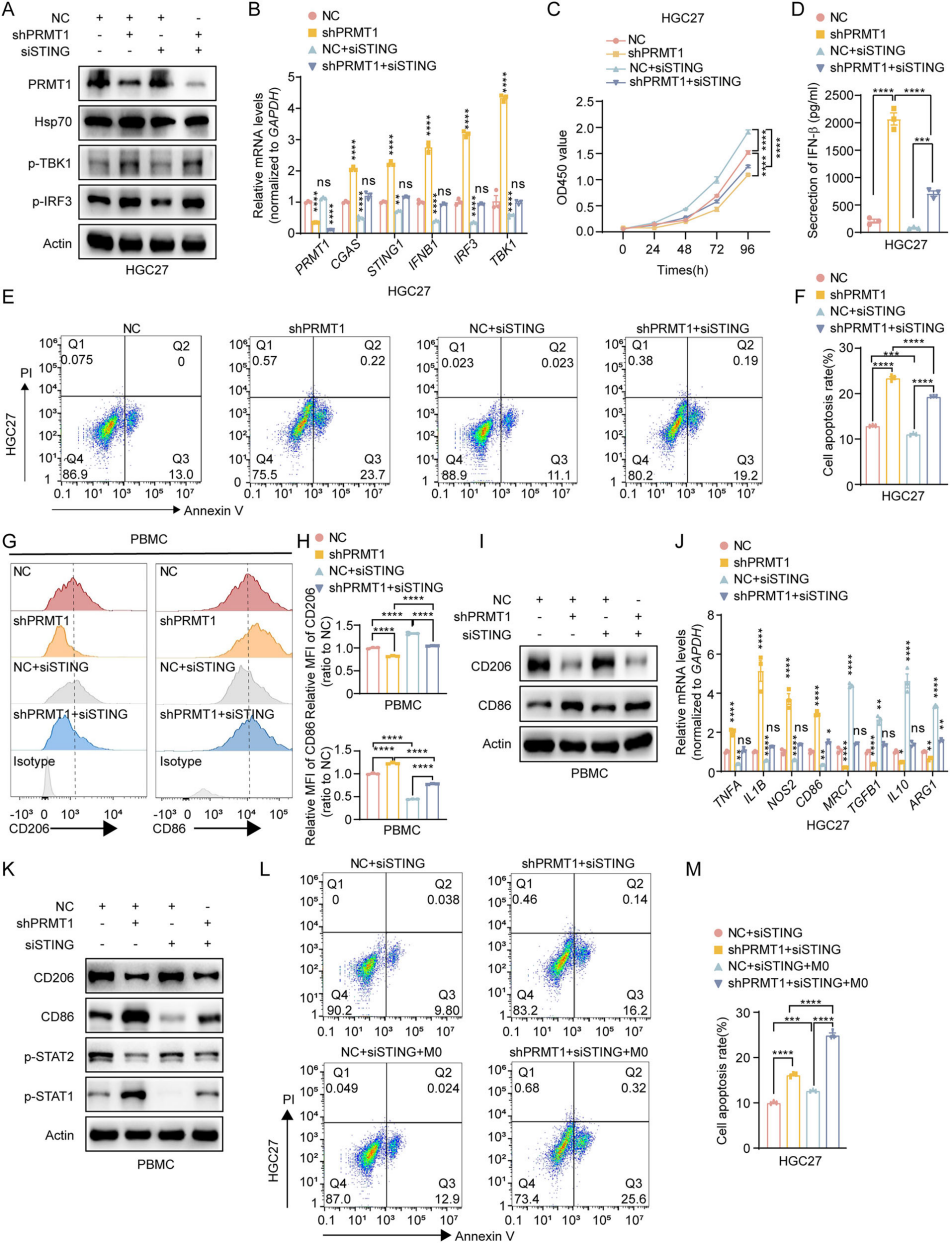
PRMT1 knockdown-induced antitumor properties and polarization of M1-like macrophages through STAT signaling were abolished after inhibition of cGAS/STING signaling. **A** Expression levels of p-TBK1 and p-IRF3 after PRMT1 or (and) STING knockdown were detected by Western blot in HGC27. **B** Expression levels of *CGAS, STING, IFN-α, TBK1, and IRF3* after PRMT1 or (and) STING knockdown were detected by qRT-PCR in HGC27. **C** The proliferation rates of HGC27 cells upon depletion of PRMT1 or (and) STING determined by CCK8 assay. **D** The expression levels of IFN-β in the secretion of HGC27 cells after depletion of PRMT1 or (and) STING detected by ELISA. **E**, **F** Flow cytometry analysis of the apoptotic cells after depletion of PRMT1 or (and) STING in HGC27 cells using the Annexin V-APC/PI staining assay (**E**). Positive Annexin V cells were displayed as a histogram (**F**). **G**, **H** PBMCs from the coculture system with HGC27 cells depleted or not depleted of PRMT1 or (and) STING and analyzed by flow cytometry with anti-CD86 and anti-CD206 antibodies (**G**). The ratio of MFI of CD86^+^ cells (**D**) and CD206^+^ cells (**H**) is shown. **I** Western blots detected protein levels of CD86 and CD206 in PBMCs from the coculture system with HGC27 cells depleted of PRMT1 or (and) STING. **J** mRNA levels of M1 and M2 macrophage markers in PBMCs from the coculture system with HGC27 cells after PRMT1 or (and) STING knockdown detected by qRT-PCR. **K** Western blots detected protein levels of CD86, CD206, p-STAT1, and p-STAT2 in PBMCs from coculture system with HGC27 cells depleted of PRMT1 or (and) STING. **L**, **M** Flow cytometry analysis of the apoptotic HGC27 cells after depletion of PRMT1 or (and) STING in HGC27 cells from the coculture system with PBMCs using the Annexin V-APC/PI staining assay (**L**). Positive Annexin V cells were displayed as a histogram (**M**). *P < 0.05, **P < 0.01, ***P < 0.001, ****P < 0.0001.

Firstly, we determined that whether PRMT1 promote GC growth by cGAS-STING pathway. The western blot results confirmed that the increased phosphorylation levels of TBK1 and IRF3 after PRMT1 knockdown were significantly reduced after the inhibition of STING (Fig. 5A and Supplementary Fig. 6A). Additionally, the increased mRNA levels of *CGAS, STING, IFN-α, TBK1 and IRF3* after PRMT1 knockdown were significantly reduced after STING inhibition (Fig. 5B and Supplementary Fig. 6B, C). Moreover, the increased apoptosis ability of GC cells after PRMT1 knockdown was significantly reduced after STING inhibition, as determined via CCK8 and flow cytometry (Fig. 5C, E, F, Supplementary Fig. 6D–I). Furthermore, the increased level of IFN-β secretion in GC cells after PRMT1 knockdown was significantly inhibited when STING expression was inhibited (Fig. 5D, Supplementary Fig. 6J, K). These findings further demonstrate that targeting PRMT1 triggers the activation of cGAS/STING signaling in GC cells.

To determine whether PRMT1 induces the polarization of tumor-infiltrating macrophages by regulating the cGAS/STING signaling pathway in GC cells, we cocultured GC cells and macrophages in vitro. Flow cytometry revealed that the phenotype of increased CD86^+^ macrophages and reduced CD206^+^ macrophages in the shPRMT1 group disappeared after STING was silenced (Fig. 5G, H and Supplementary Fig. 7A, B). The decrease in the expression of M2-like macrophage markers slightly improved, whereas the increase in the expression of M1-like markers significantly weakened after the combined inhibition of PRMT1 and STING (Fig. 5I, J and Supplementary Fig. 7C, D). Moreover, the phosphorylation of STAT1 and STAT2 in macrophages also changed when STING was inhibited (Fig. 5K and Supplementary Fig. 7E). In addition, we examined the proliferation of GC cells cocultured with or without macrophages, and the results demonstrated that, compared with non-coculture conditions, the inhibition of STING reduced the apoptotic capacity of GC cells and attenuated the shPRMT1-induced suppression of GC cell proliferation, with macrophage coculture further amplifying these effects (Fig. 5L, M and Supplementary Fig. 7F, G). These results suggest that PRMT1 deficiency enhances the cGAS/STING pathway in GC cells, which producing IFNβ, which activates STAT1 signaling in macrophages and induces M1 macrophage polarization, ultimately inhibiting GC tumorigenesis.

### The H151 STING inhibitor enhances the anticancer effect in PRMT1-deficient GC

Considering that the nu/nu nude mice used in the above experiments had congenital defects in thymus development, resulting in few mature T lymphocytes but that other immune cells were abundant [19], we used two xenograft tumor mouse models (mouse MFC GC cells implanted in C57BL/6 mice and human HGC27 cells implanted in mice) to explore the actual immune response in GC.

**Fig. 6:**
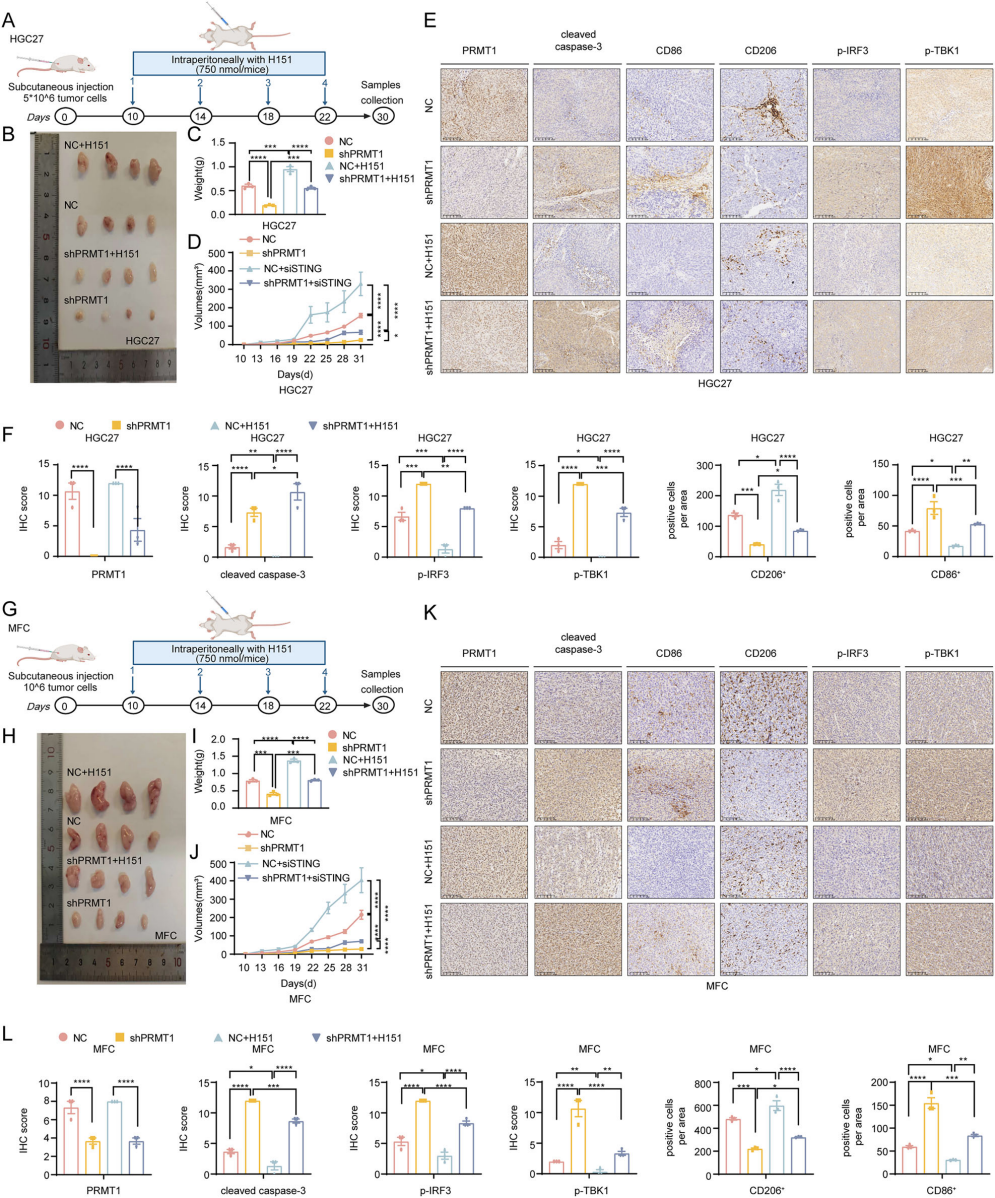
STING inhibitor H151 enhanced the anticancer effect in PRMT1-deficient GC in vivo. **A** Schematic of the H151 treatment schedule in the xenograft gastric cancer model (injection of HGC27 cells with control group and PRMT1 knockdown group). **B**−**D** Tumor macroscopical images (**B**), tumor weights (**C**) and volumes (**D**) at the end of the experiments in the shPRMT1 and NC groups with or without H151 treatment. **E** Representative microphotographs of IHC staining of PRMT1, cleaved caspase-3, p-TBK1, p-IRF3, CD86^+^, and CD206^+^ cells in the upper 4 groups. **F** H score of PRMT1, cleaved caspase-3, p-TBK1, and p-IRF3, and positive cells per area of CD86^+^ and CD206^+^ cells in the upper 4 groups. Magnification: 400 ×. **G** Schematic of the H151 treatment schedule in the xenograft gastric cancer model (injection of MFC cells with control group and PRMT1 knockdown group). **H**−**J** Tumor macroscopical images (**H**), tumor weights (**I**), and volumes (**J**) at the end of the experiments in the shPRMT1 and NC groups with or without H151 treatment. **K** Representative microphotographs of IHC staining of PRMT1, cleaved caspase-3, p-TBK1, p-IRF3, CD86^+^, and CD206^+^ cells in the upper 4 groups. Magnification: 400 ×. **L** H score of PRMT1, cleaved caspase-3, p-TBK1, and p-IRF3, and positive cells per area of CD86^+^ and CD206^+^ cells in the upper 4 groups. ^*^P < 0.05, ^**^P < 0.01, ^***^P < 0.001, ^****^P < 0.0001.

In both models, we evaluated whether targeting STING promotes anticancer effects following PRMT1 inhibition in GC. At 10, 14, 18 and 22 days after the injection with cancer cells, the mice were treated with an intratumoral H151 injection (Fig. 6A, G). Although the PRMT1 knockdown group alone showed good anticancer effects, the inhibited tumor growth was restored after the combined inhibition of STING (Fig. 6B–D, H–J). As shown in Fig. 6, the increased levels of cleaved caspase3, p-TBK1 and p-IRF3 in the shPRMT1 group decreased after STING was inhibited (Fig. 6E, F, K, L). Overall, we provide robust data demonstrating that targeting STING inhibits the anticancer effects of inhibiting PRMT1 in GC.

Moreover, we performed IHC staining and flow cytometry on markers of macrophage infiltration in the above two models. Compared with that in the control group, increased M1-like macrophage infiltration in the shPRMT1 group was decreased after STING was inhibited, whereas decreased M2-like macrophage infiltration in the shPRMT1 group was increased (Fig. 6E, F, K, L). Furthermore, flow cytometry revealed that the phenotype of increased CD86^+^ macrophages and reduced CD206^+^ macrophages in the shPRMT1 group disappeared after H151 injection in the C57BL/6 mouse group (Supplementary Fig. 7H, I). These findings demonstrated in vitro that the cGAS/STING signaling pathway activated by PRMT1 knockdown contributes to the polarization of M1-like macrophages in the TME and attenuates tumor progression in GC.

To investigate the effects of PRMT1 methyltransferase activity and macrophage polarization on gastric cancer progression in vivo, we conducted experiments using the PRMT1 enzymatic activity inhibitor MS023 and the macrophage-depleting agent clodronate liposomes. We observed a significant increase in the proliferation of subcutaneous tumor tissues in mice after macrophage depletion (Supplementary Fig. 8). This result indicates that the pro-tumorigenic effect of PRMT1 on the in vivo growth of gastric cancer cells is dependent on macrophages.

**Fig. 7:**
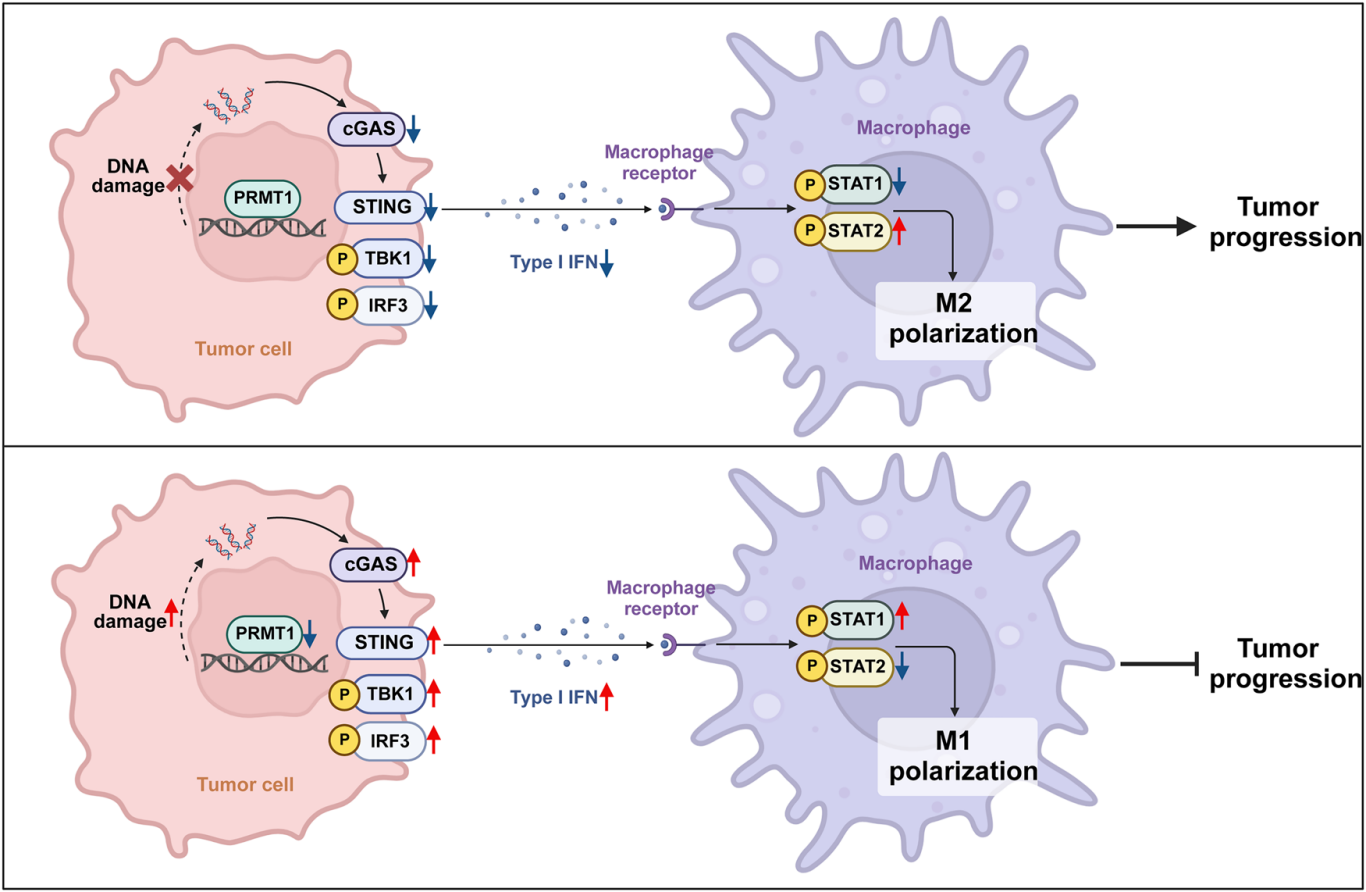
Schematic illustration of tumor-derived PRMT1 suppresses macrophage antitumor activity by inhibiting cGAS/STING signaling in gastric cancer cells. PRMT1 knockdown inhibited the cGAS/STING pathway in GC, which produced type I IFNs to promote the polarization of M1-like macrophages in the tumor microenvironment (Created with BioRender.com).

In summary, our work revealed that PRMT1 knockdown disrupts immunosuppressive conditions in GC through increased DNA damage, thereby activating cGAS/STING signaling and enabling the polarization of M1-like macrophages in the TME to trigger antitumor immune responses (Fig. 7).

## Discussion

Recent studies have shown that PRMT1 acts as an epigenetic transcription regulator by activating gene transcription, thereby regulating the occurrence and development of a variety of tumors, including breast cancer, hepatocellular carcinoma, and colorectal cancer [20–23]. However, the study of the function of PRMT1 in GC has improved slowly. Only a few studies have suggested that PRMT1 is highly expressed in GC and promotes tumor progression [24]. Recently, Wang reported that PRMT1 activates related pathways by recruiting MLXIP to the promoter region of β-catenin and thus plays a role in promoting tumor cell growth and metastasis [25]. Our study also confirmed the expression level of PRMT1 is higher in human GC tissues than in normal tissues, and this was associated with poor prognosis. Further analysis revealed the tumor-suppressive function of PRMT1 after its knockdown in vivo and in vitro. In summary, PRMT1 can be used as a valuable indicator in the diagnosis and prediction of GC.

dsDNA in tumor cells can be sensed through the cGAS/STING pathway. Activation of cGAS/STING leads to the phosphorylation of TBK1 and IRF3, resulting in the production of type I IFN and triggering antitumor effects [26, 27]. Wu recently demonstrated that HER2 signaling disrupts STING signal activation by suppressing the TBK1/IRF3 axis [28]. It was also found that targeting MUS81 can activate cGAS/STING signaling, thereby enhancing the anticancer effect of WEE1 in GC [29]. Moreover, in human GC cells, STING expression in tumor tissue is significantly reduced in a TNM stage-dependent manner, and STING knockdown promotes GC cell survival [30, 31]. However, in another study by Miao, the STING protein was found to be more highly expressed in malignant tissues than in normal tissues, and high expression of STING predicted lower survival rates in patients with GC [32]. The reasons for this discrepancy remain unclear. Our experiments confirmed that the proliferation ability of GC cells was significantly enhanced after the inhibition of STING. However, we lack clinical studies with large sample sizes to confirm that low STING expression predicts lower survival in patients with GC.

PRMT inhibition is known to induce the production of misspliced mRNAs containing retained introns, which further promote dsRNA formation and the activation of the type I interferon response in tumor cells. For example, PRMT2/4-mediated arginine methylation is pivotal for transcription, DNA repair, and tumor growth [33]. In this study, we found that PRMT1 deletion is increased through DNA damage, leading to the accumulation of endogenous dsDNA. This accumulation of dsDNA activates the cGAS/STING pathway, resulting in significantly increased phosphorylation levels of TBK1 and IRF3. This activation also results in increased secretion of type I interferons, thus promoting the progression of GC. Additionally, combined inhibition of PRMT1 and STING expression in GC resulted in a slight recovery of tumor growth. These results suggest that the expression level of STING should be evaluated concurrently when targeting PRMT1 for GC therapy. However, the potential epigenetic modification by which PRMT1 downregulates type I interferon secretion warrants further exploration.

In fact, PRMTs play important roles in tumor immunomodulatory functions. PRMT5 has been reported to suppress antitumor T-cell immunity by methylating IFI16 to reduce dsDNA-induced interferon responses [34]. Tao reported that PRMT1 acts as an inhibitor of CD8^+^ T-cell recruitment and activation in melanoma and that deletion of PRMT1 reduces the abundance of H3K27ac and H4R3me2a in the Dnmt1 enhancer region, thereby reducing Dnmt1 expression in melanoma cells [35]. Here, we revealed a novel link between the dsDNA-induced antitumor ability of PRMT1 and macrophages in GC. In this study, RNA-seq and TCGA data revealed that PRMT1 expression was associated with the infiltration of immune cells, including B cells, regulatory T cells, macrophages, and mast cells. High expression of PRMT1 is associated with macrophage regulation, wound healing, lymphocyte infiltration and matrix fraction. Therefore, we hypothesize that PRMT1 plays an important role in the maintenance of the GC immune microenvironment.

Tumor-associated macrophages are an important part of the TME [36]. Highly heterogeneous and plastic TAMs rapidly adapt to changes in the TME, polarizing into M1-polarized macrophages (inflammatory, activated type) or M2-polarized macrophages (alternately activated, immunosuppressive type) [36]. Previous studies have confirmed that there is a significant negative correlation between macrophage infiltration and patient survival in TCGA GC data [37]. In recent years, an increasing number of researchers have attempted to alter the immunosuppressive status of GC by adjusting the polarization of the TAM phenotype. Zheng demonstrated that M2-polarized macrophages promote the resistance of GC cells to cisplatin and that M2 macrophage-derived exosomal miR-21 reduces chemical sensitivity to cisplatin [38]. Moreover, CSF-1 secreted by GC cells promotes TAM recruitment, which is thought to contribute to chemotherapy resistance [39]. In our studies, there was an increase in the number of M1-like macrophages with antitumor properties after PRMT1 knockdown. Coculture experiments revealed that PRMT1 knockdown promoted the polarization of M1-like macrophages. Notably, correlation analysis of human GC microarrays further revealed that PRMT1 was negatively correlated with M1-like TAMs and positively correlated with M2-like TAMs. These findings can provide a direct reference for further antitumor research and immunotherapy in patients with GC.

Importantly, until now, we have not been able to determine which factors are involved in the polarization of M1-like TAMs after PRMT1 knockdown. Previous studies have confirmed that the cGAS/STING pathway is also considered to be an important part of activating the antitumor immune response [40]. STING signaling in tumor cells is significantly correlated with intratumor immune cell infiltration and antigen presentation [41]. PMMA nanoplastics activate the cGAS/STING pathway, promote the natural immune response, upregulate inflammatory markers, and induce gastritis [42]. Wu recently demonstrated the direct interaction between HER2, Akt, and TBK1 in GC and that blocking the HER2/Akt cascade enhances STING signaling-mediated antitumor immunity [28]. In addition, STING activation in GC not only promotes the differentiation of macrophages into proinflammatory subtypes but also induces the apoptosis of GC cells through the activation of the IL6R-JAK-IL24 pathway [32]. However, it is unclear whether PRMT1 influences macrophage polarization through cGAS/STING signaling in GC. Here, we revealed a novel association between PRMT1 inhibition of tumor macrophage polarization and cGAS/STING in a GC model. Increased dsDNA is produced by the cGAS/STING pathway, which ultimately triggers the type I interferon pathway, leading to the recruitment and activation of M1 macrophages in tumors and the inhibition of GC growth. Our study strongly supports the specific role of PRMT1 in interferon-related antitumor immunity.

Inflammation plays a dual role in chronic diseases such as tumors [43]. Chronic inflammation can lead to the recruitment of immunosuppressive cells such as regulatory T cells, myeloid-derived suppressor cells (MDSCs), and M2-like tumor-associated macrophages [44]. These cells secrete anti-inflammatory cytokines, inhibit cytotoxic T cell function, and promote tissue remodeling, all of which create a tumor-permissive microenvironment that facilitates immune evasion and cancer progression. However, IFN-β, a key type I interferon, is also well-documented to exert potent anti-tumor effects. It promotes dendritic cell maturation, enhances antigen presentation, activates CD8^+^ T cells, and recruits NK cells, thereby stimulating effective anti-tumor immunity [45]. In our study, PRMT1 knockdown induced DNA damage and cytosolic dsDNA accumulation, leading to cGAS/STING pathway activation and subsequent IFN-β upregulation. This suggests that PRMT1 deficiency may enhance tumor immunogenicity and trigger an immune-activating response. Thus, our findings align with existing literature on the pro-immunogenic role of cGAS/STING–IFN signaling and provide mechanistic insight into how PRMT1 loss may enhance anti-tumor immunity without contradicting the broader understanding of inflammation’s dual roles in cancer.

The STAT signaling pathway is a more central pathway in the process of macrophage polarization, especially the balance between STAT1 and STAT2 activation [46]. Specifically, the activation of the STAT1 pathway induces M1 polarization of macrophages, exerting inflammatory functions and cytotoxic effects, whereas the activation of the STAT2 pathway mediates M2 polarization of macrophages, inhibiting tumor immunity and promoting the progression of GC [47, 48]. These findings confirmed that, after PRMT1 knockdown, the activation of the STAT signaling pathway in cocultured macrophages in GC cells was affected, resulting in increased M2 polarization and decreased M1 polarization of macrophages. Furthermore, the combined inhibition of PRMT1 and STING expression confirmed that PRMT1 in GC cells affects the STAT signaling pathway in macrophages through the cGAS/STING signaling pathway, thus altering the polarization of macrophages.

Overall, our study revealed that PRMT1 expression is increased in human GC cells and that PRMT1 knockdown can prevent the growth of GC tumors in vivo. In addition, PRMT1 knockdown led to changes in the STAT signaling pathway in the GC TME, invasive M1-like TAM polarization, an enhanced antitumor immune response, and inhibited tumor progression in GC. To our knowledge, this is the first study to elucidate the regulatory role of PRMT1 in the immune microenvironment of GC and the relationship between mononuclear/macrophage recruitment and M1-like TAM polarization after PRMT1 knockdown. These findings suggest that PRMT1 may be a useful target for specific epigenetic therapies for GC cells. Our study provides a practical application for a new PRMT1 small molecule inhibitor that may alter the TME and transform GC from “cold tumors” to “hot tumors”. Therefore, PRMT1 plays an important role in GC development and is a promising target for GC treatment.

## Supplementary information

Fig. S1: PRMT1 overexpression was linked to poor prognosis in GC patients and promoted the progression of HGC27 cells.

Fig. S2: Cancer promoting functions of PRMT1 in GC.

Fig. S3: PRMT1 knockdown triggered an antitumor response via the activation of cGAS/STING signaling.

Fig. S4: PRMT1 amplifies cytosolic DNA accumulation and promotes innate immune activation in GC.

Fig. S5: PRMT1 enzyme activity - induced antitumor properties leads to increased infiltration and polarization of M1-like TAMs in GC to improve antitumor ability.

Fig. S6: PRMT1 knockdown-induced antitumor properties were abolished after inhibition of cGAS/STING signaling.

Fig. S7: PRMT1 knockdown-induced antitumor properties and polarization of M1-like macrophages through STAT signaling were abolished after inhibition of cGAS/STING signaling.

Fig. S8: PRMT1 inhibitor MS023 and macrophage-depleting agent clodronate liposomes on mice affects xenograft gastric cancer growth in vivo.

The supplementary tables.

Original western blots of this manuscript.

Supplementary figure legend

## Data Availability

The datasets generated during and/or analysed during the current study are available from the corresponding author on reasonable request.
