# Supplementary figures and images for: Tumor-derived PRMT1 suppresses macrophage antitumor activity by inhibiting cGAS/STING signaling in gastric cancer cells
Source: Cell Death Dis. 2025 Aug 26;16(1):649. doi: 10.1038/s41419-025-07960-y (PMC12381180; doi:10.1038/s41419-025-07960-y)

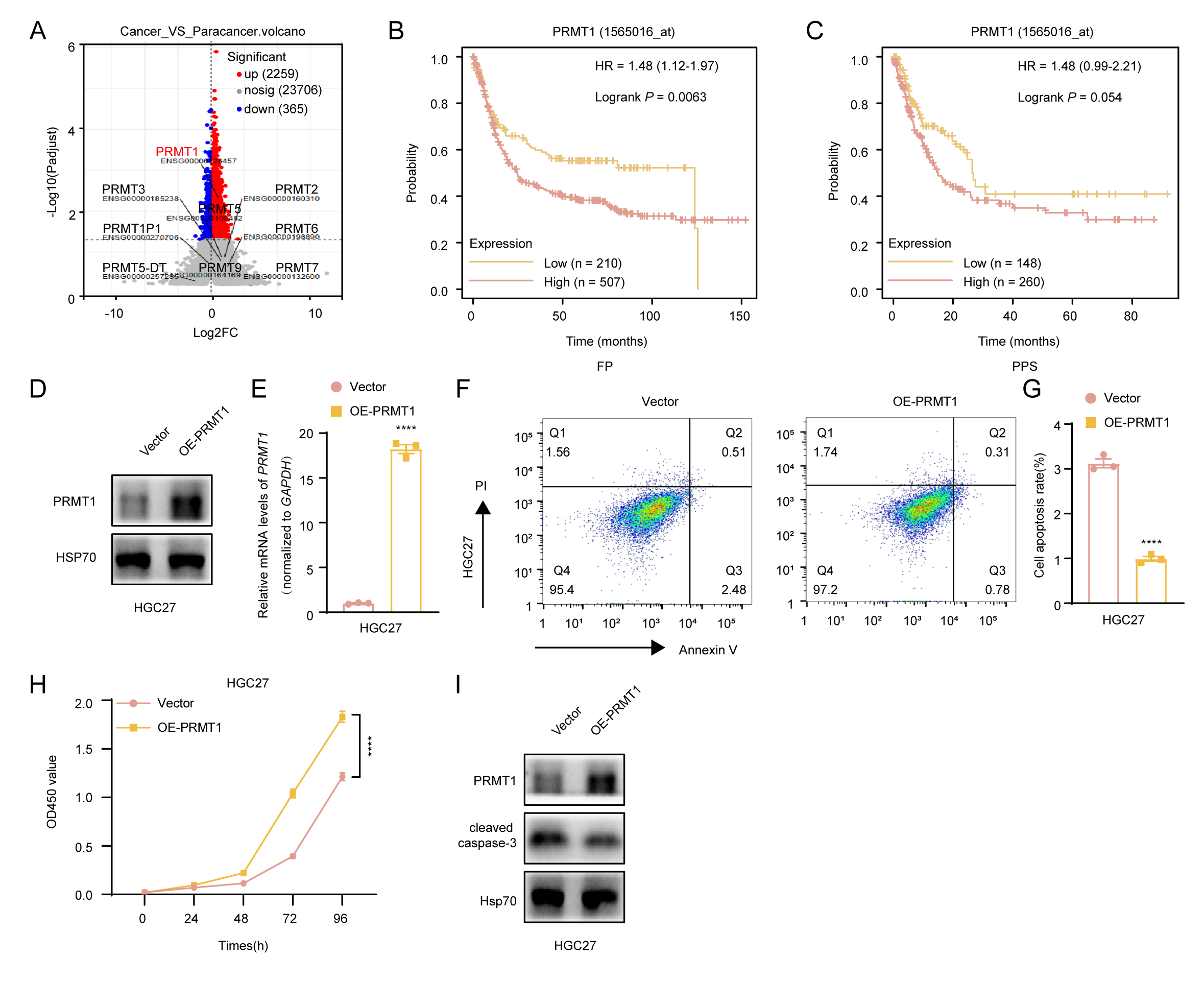

Supplement: Supplementary file 1 — Fig. S1: PRMT1 overexpression was linked to poor prognosis in GC patients and promoted the progression of HGC27 cells. [file 41419_2025_7960_MOESM1_ESM.tif]

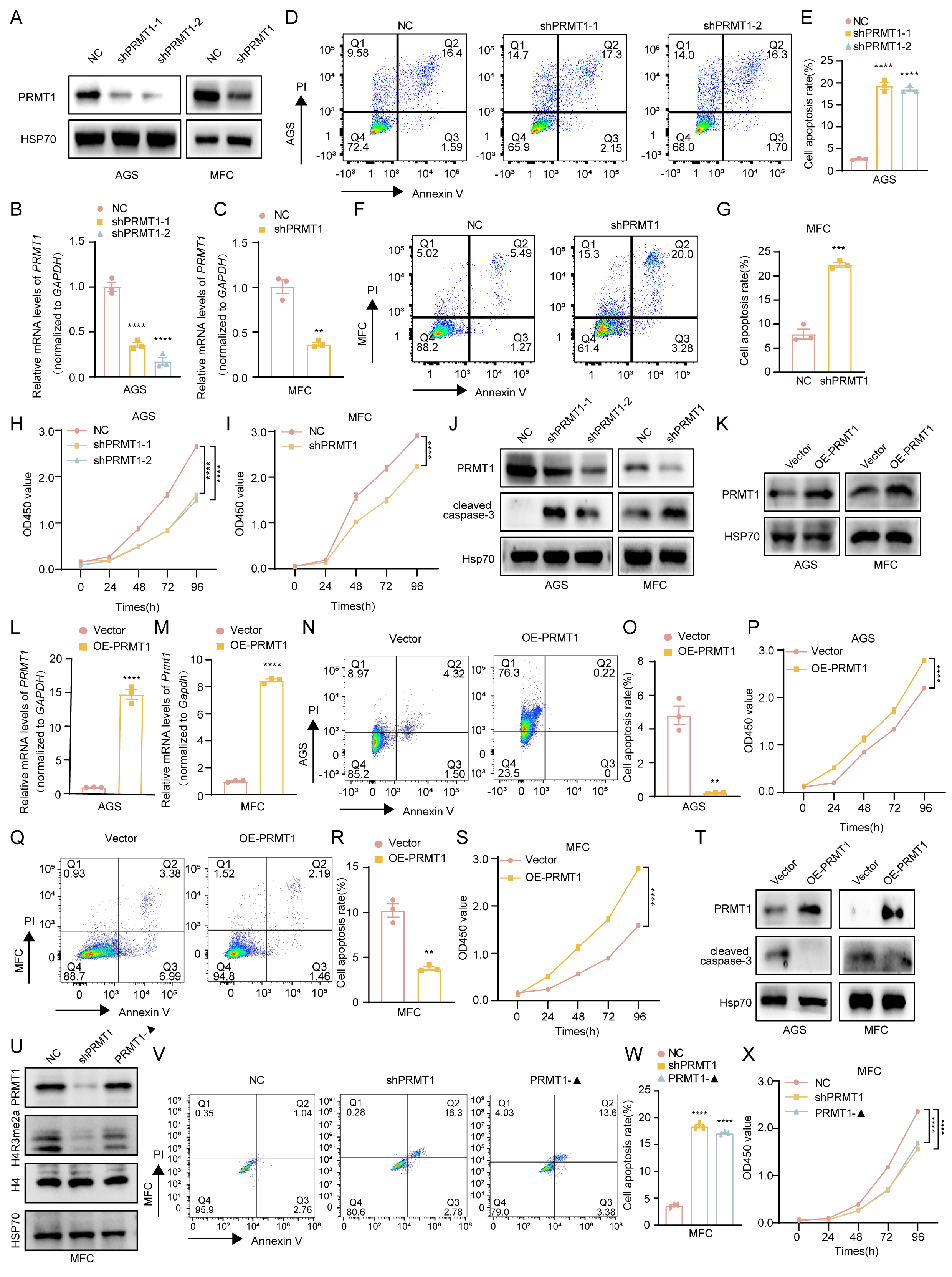

Supplement: Supplementary file 2 — Fig. S2: Cancer promoting functions of PRMT1 in GC. [file 41419_2025_7960_MOESM2_ESM.tif]

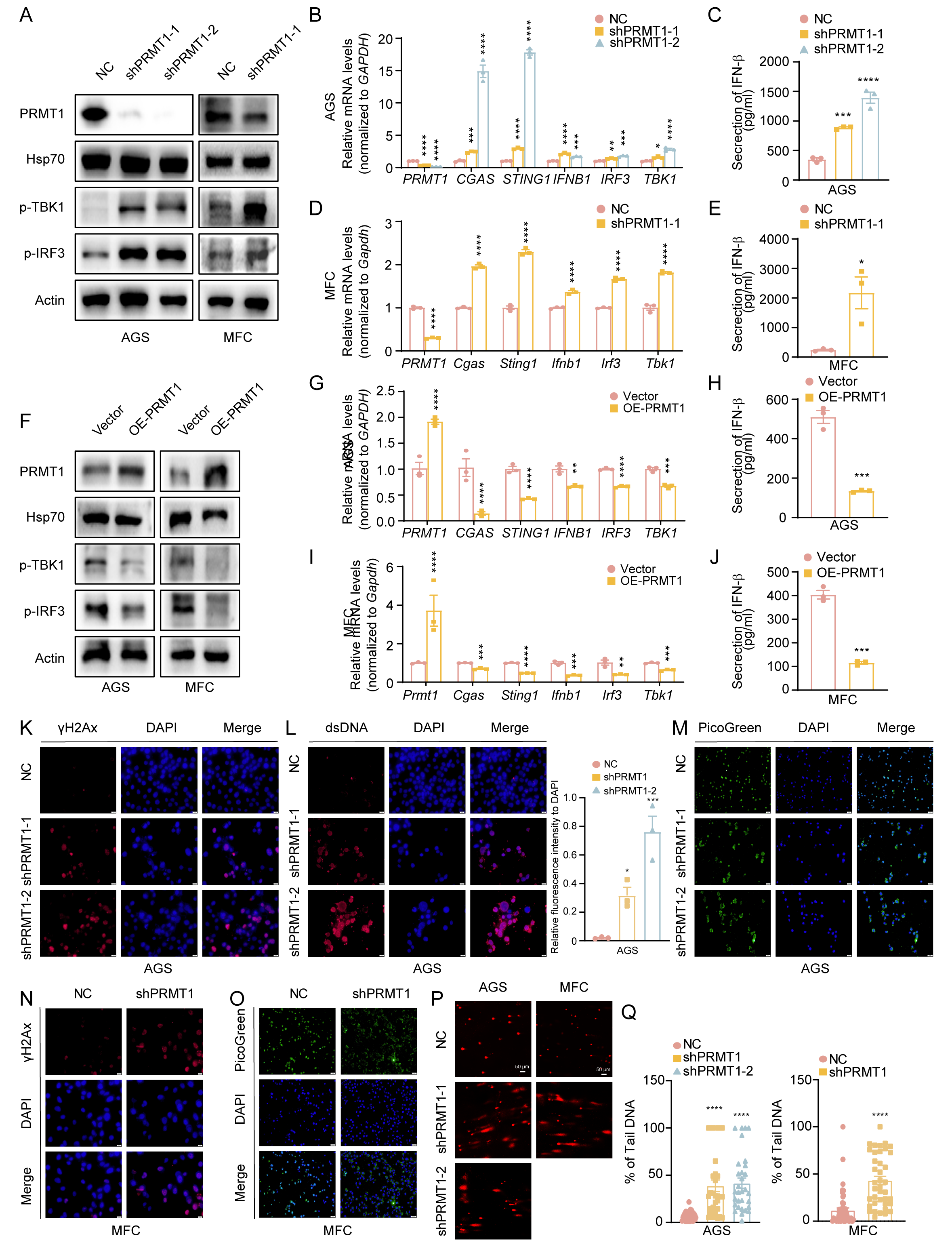

Supplement: Supplementary file 3 — Fig. S3: PRMT1 knockdown triggered an antitumor response via the activation of cGAS/STING signaling. [file 41419_2025_7960_MOESM3_ESM.tif]

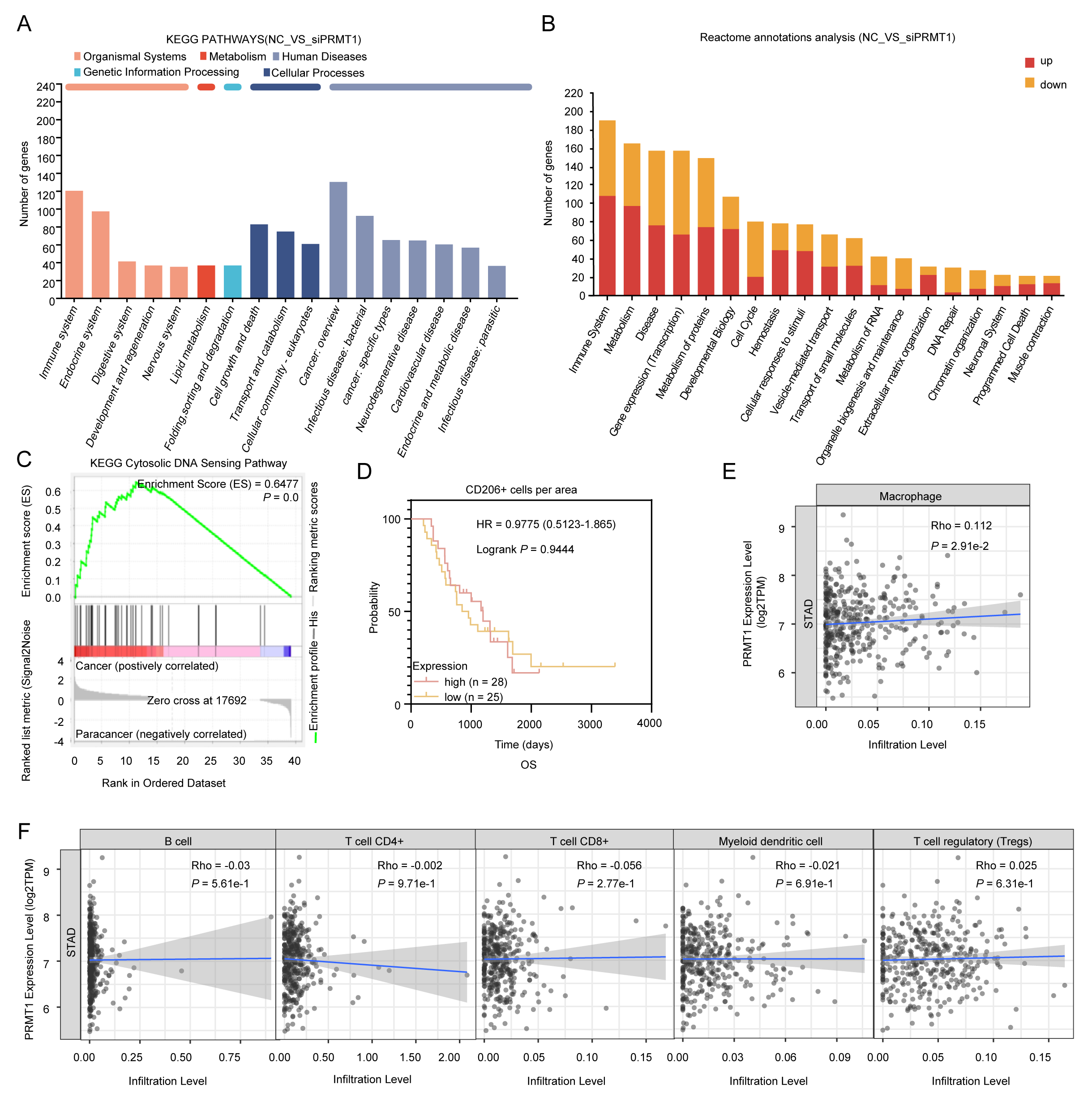

Supplement: Supplementary file 4 — Fig. S4: PRMT1 amplifies cytosolic DNA accumulation and promotes innate immune activation in GC. [file 41419_2025_7960_MOESM4_ESM.tif]

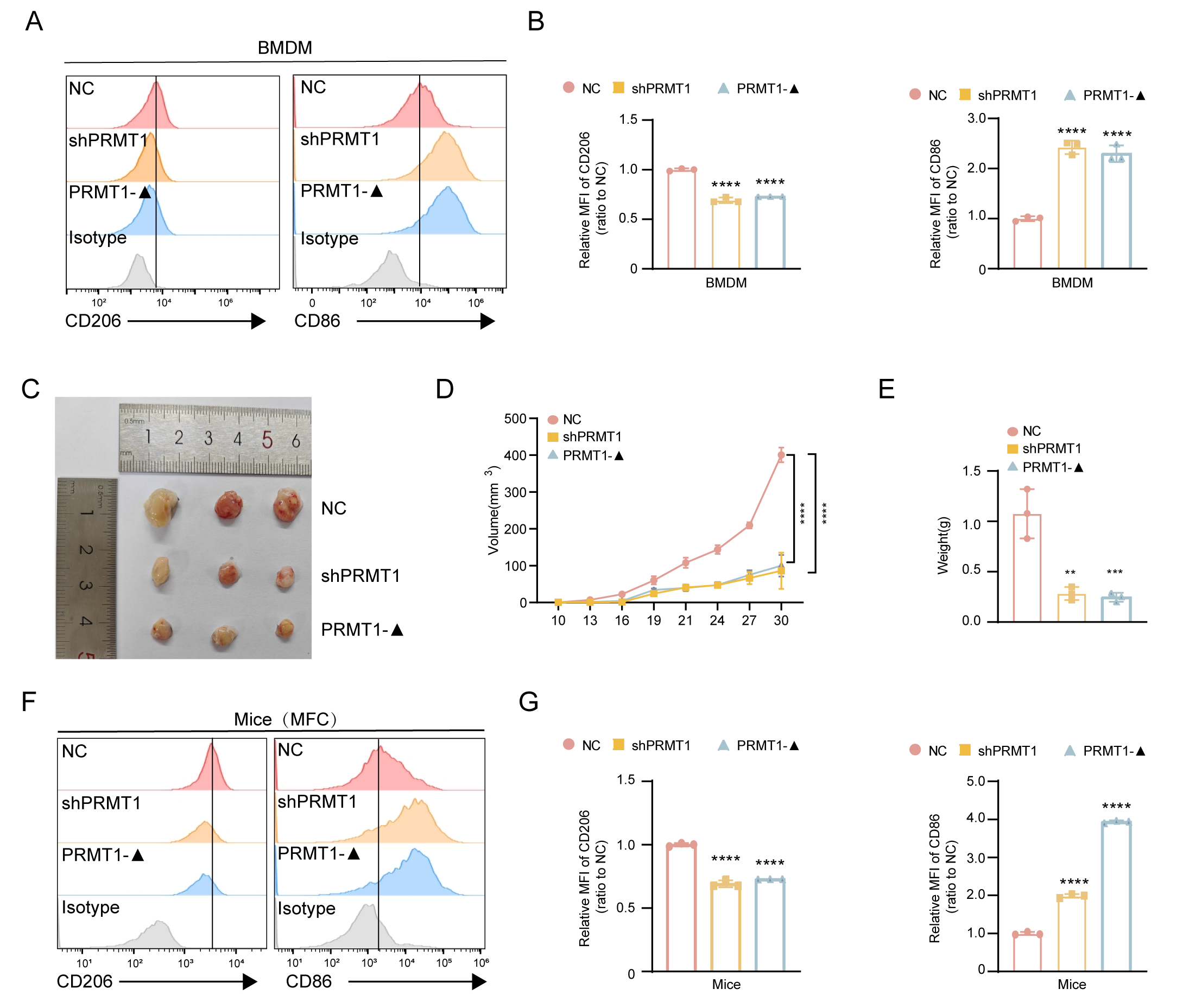

Supplement: Supplementary file 5 — Fig. S5: PRMT1 enzyme activity - induced antitumor properties leads to increased infiltration and polarization of M1-like TAMs in GC to improve antitumor ability. [file 41419_2025_7960_MOESM5_ESM.tif]

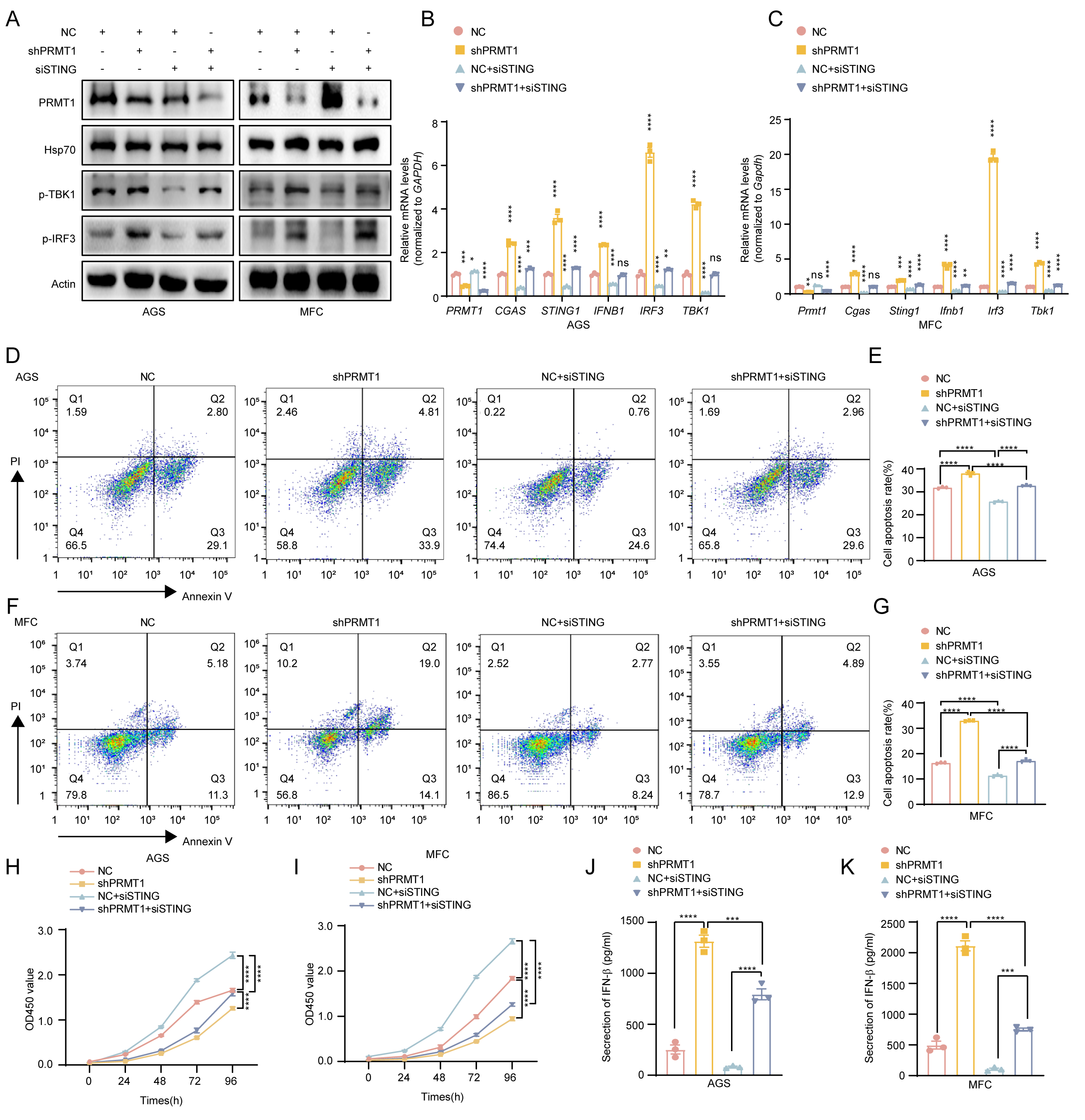

Supplement: Supplementary file 6 — Fig. S6: PRMT1 knockdown-induced antitumor properties were abolished after inhibition of cGAS/STING signaling. [file 41419_2025_7960_MOESM6_ESM.tif]

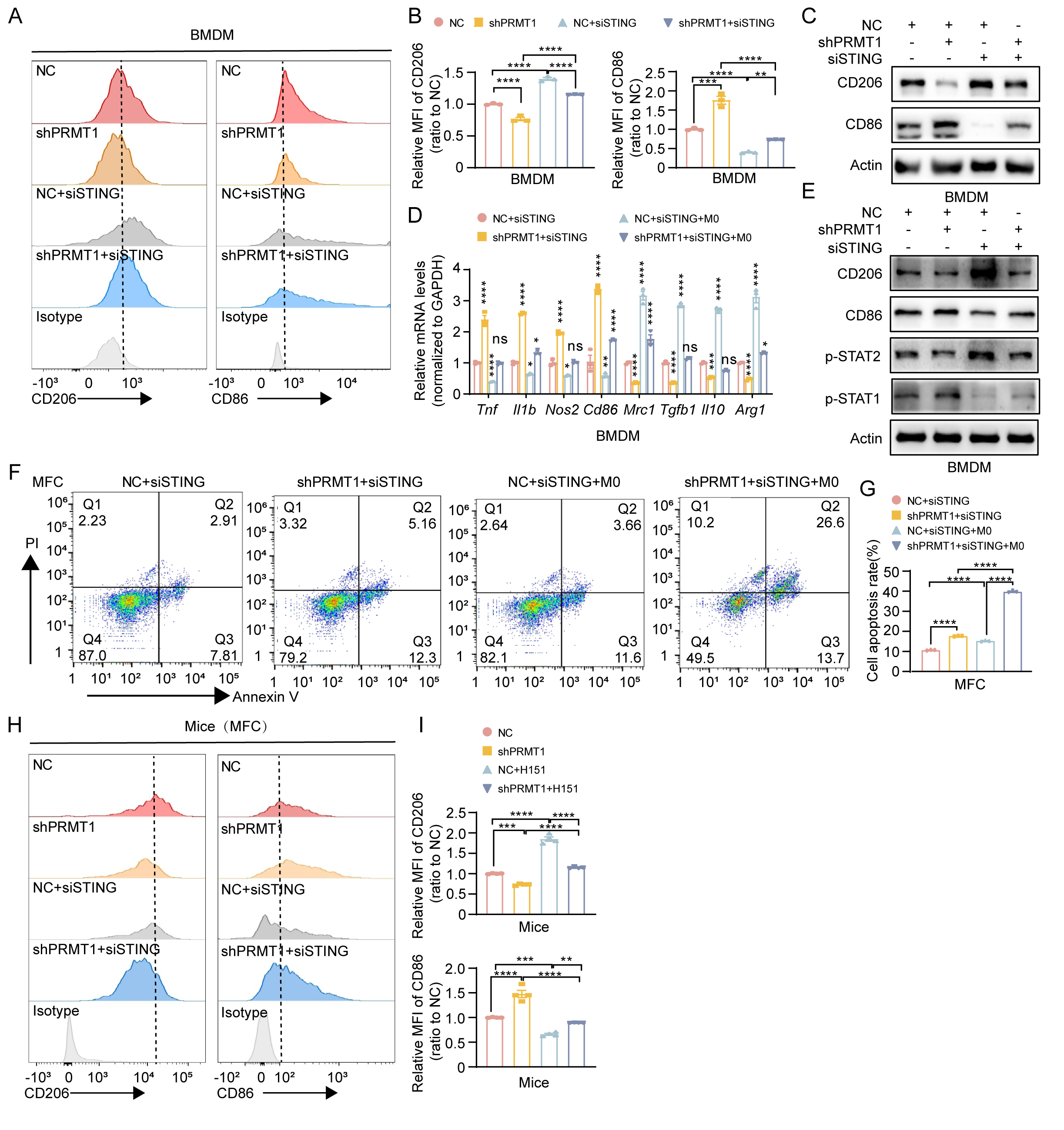

Supplement: Supplementary file 7 — Fig. S7: PRMT1 knockdown-induced antitumor properties and polarization of M1-like macrophages through STAT signaling were abolished after inhibition of cGAS/STING signaling. [file 41419_2025_7960_MOESM7_ESM.tif]

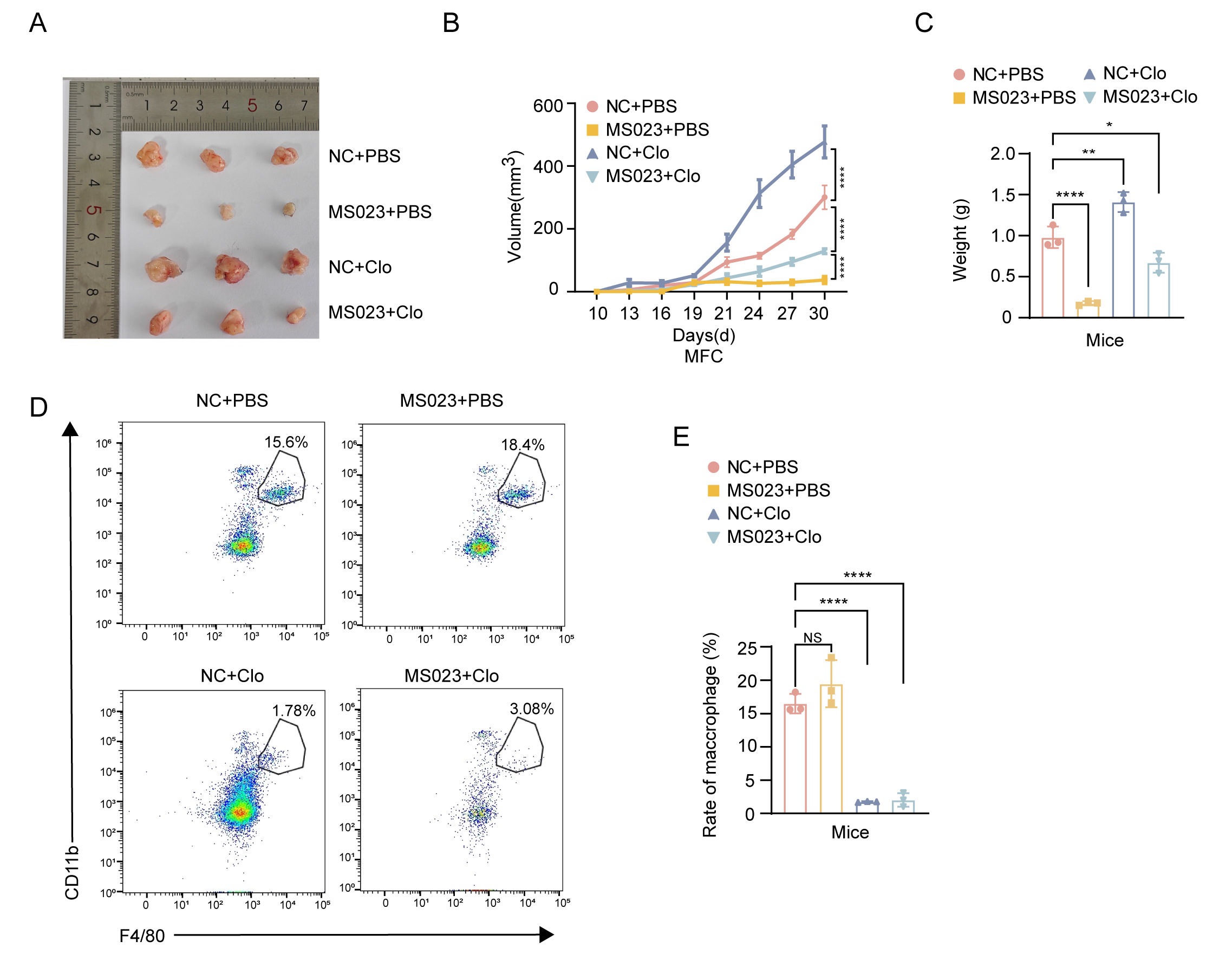

Supplement: Supplementary file 8 — Fig. S8: PRMT1 inhibitor MS023 and macrophage-depleting agent clodronate liposomes on mice affects xenograft gastric cancer growth in vivo. [file 41419_2025_7960_MOESM8_ESM.tif]
