# Supplementary material for: Tumor-derived PRMT1 suppresses macrophage antitumor activity by inhibiting cGAS/STING signaling in gastric cancer cells
Source: Cell Death Dis. 2025 Aug 26;16(1):649. doi: 10.1038/s41419-025-07960-y (PMC12381180; doi:10.1038/s41419-025-07960-y)
Supplement: Supplementary file 9 — The supplementary tables. [file 41419_2025_7960_MOESM9_ESM.docx]

**Table S1. The list of sequences for Viruses**

| **Names** | **Sequence (5’-3’)** |
| --- | --- |
| shPRMT1-1(human) | GTTGTAGTCTTTGTACTGCC |
| shPRMT1-2(human) | GCCGGCAGTACAAAGACTACAA |
| siPRMT1(human) | CGUCAAAGCCAACAAGUUA |
| siSTING(human) | GCATTACAACAACCTGCTA |
| shPRMT1(mouse) | CGCAACTCCATGTTTCACAAT |
| siSTING(mouse) | GGATCCGAATGTTCAATCA |
| PRMT1-▲ | GGTGGTGCTGGACGTCATCCTCTGCATGTTTGC |

**Table S2. The list of primer sequences for qRT-PCR.**

| **Names** | **Sequence (5’-3’)** |
| --- | --- |
| CGAS-F | ACATGGCGGCTATCCTTCTCT |
| CGAS-R | GGGTTCTGGGTACATACGTGAAA |
| STING1-F | AGCATTACAACAACCTGCTACG |
| STING1-R | GTTGGGGTCAGCCATACTCAG |
| IFNB1-F | GTCACTGTGCCTGGACCATAG |
| IFNB1-R | GTTTCGGAGGTAACCTGTAAGTC |
| IRF3-F | TCCCACTCCCTTCCCAAACCT |
| IRF3-R | AGCGTCCTGTCTCCCACTTCG |
| TBK1-F | TGGCAAGGAACTGGAGAATG |
| TBK1-R | TCCAGGTCAGGCTCATCTTC |
| Cgas-F | ACCGGACAAGCTAAAGAAGGTGCT |
| Cgas-R | GCAGCAGGCGTTCCACAACTTTAT |
| Sting1-F | GGTCACCGCTCCAAATATGTAG |
| Sting1-R | CAGTAGTCCAAGTTCGTGCGA |
| Ifnb1-F | CAGCTCCAAGAAAGGACGAAC |
| Ifnb1-R | GGCAGTGTAACTCTTCTGCAT |
| Irf3-F | GAGAGCCGAACGAGGTTCAG |
| Irf3-R | CTTCCAGGTTGACACGTCCG |
| Tbk1-F | ACTGGTGATCTCTATGCTGTCA |
| Tbk1-R | TTCTGGAAGTCCATACGCATTG |
| MRC1-F | GGGTTGCTATCACTCTCTATGC |
| MRC1-R | TTTCTTGTCTGTTGCCGTAGTT |
| CD86-F | CTGCTCATCTATACACGGTTACC |
| CD86-R | GGAAACGTCGTACAGTTCTGTG |
| TNFA-F | TCTCGAACCCCGAGTGACAA |
| TNFA-R | TGAAGAGGACCTGGGAGTAG |
| TGFB1-F | AAGGACCTCGGCTGGAAGTGC |
| TGFB1-R | CCGGGTTATGCTGGTTGTA |
| IL1B-F | TGAACTGAAAGCTCTCCACC |
| IL1B-R | CTGATGTACCAGTTGGGGAA |
| IL10-F | ACCAAGACCCAGACATCA |
| IL10-R | TTCACAGGGAAGAAATCG |
| NOS2-F | CAGGACTCACAGCCTTTGGAC |
| NOS2-R | TGGATGTCGGACTTTGTAGATTC |
| ARG1-F | TGGACAGACTAGGAATTGGCA |
| ARG1-R | CCAGTCCGTCAACATCAAAACT |
| PRMT1-F | CTTTGACTCCTACGCACACTT |
| PRMT1-R | GTGCCGGTTATGAAACATGGA |
| GAPDH-F | GAAGGTGAAGGTCGGAG |
| GAPDH-R | GAAGATGGTGATGGGATTTC |
| Mrc1-F | ATGGATTGCCCTGAACAGCA |
| Mrc1-R | CTCGTCAGCACCCCAGTTAG |
| Cd86-F | GGTGGCCTTTTTGACACTCTC |
| Cd86-R | TGAGGTAGAGGTAGGAGGATCTT |
| Tnf-F | GACGTGGAACTGGCAGAAGAG |
| Tnf-R | TTGGTGGTTTGTGAGTGTGAG |
| Tgfb1-F | CTCCCGTGGCTTCTAGTGC |
| Tgfb1-R | GCCTTAGTTTGGACAGGATCTG |
| Il1b-F | CCTTCCAGGATGAGGACATGA |
| Il1b-R | TGAGTCACAGAGGATGGGCTC |
| Il10-F | GCTCTTACTGACTGGCATGAG |
| Il10-R | CGCAGCTCTAGGAGCATGTG |
| Nos2-F | GAGCCAGTCCTCTTTGCT |
| Nos2-R | CAACCTTGGTGTTGAAGGCG |
| Arg1-F | ACATTGGCTTGCGAGACGTA |
| Arg1-R | ATCACCTTGCCAATCCCCAG |
| Prmt1-F | TACTACTTTGACTCCTATGCCCA |
| Prmt1-R | ATGCCGATTGTGAAACATGGA |
| Gapdh-F | GGTGAAGGTCGGTGTGAACG |
| Gapdh-R | CTCGCTCCTGGAAGATGGTG |
