# Supplementary material for: Tumor-derived PRMT1 suppresses macrophage antitumor activity by inhibiting cGAS/STING signaling in gastric cancer cells
Source: Cell Death Dis. 2025 Aug 26;16(1):649. doi: 10.1038/s41419-025-07960-y (PMC12381180; doi:10.1038/s41419-025-07960-y)

Fig1.H

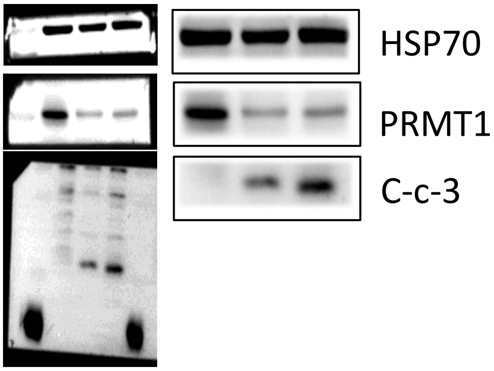

Fig1.M

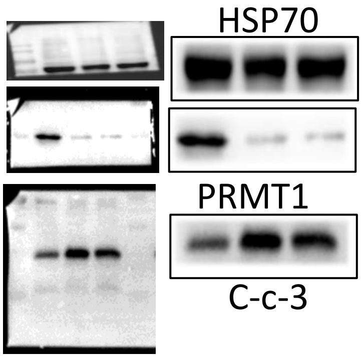

Fig2.I

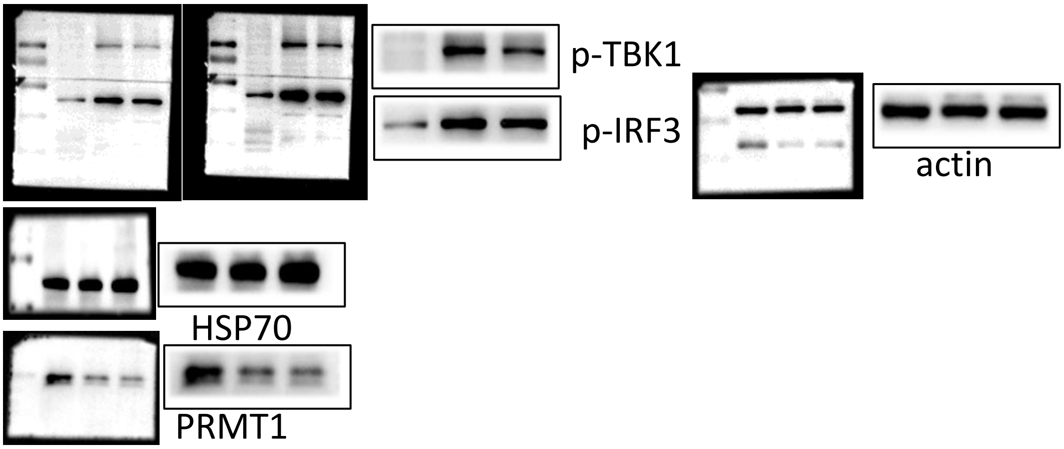

Fig2.J

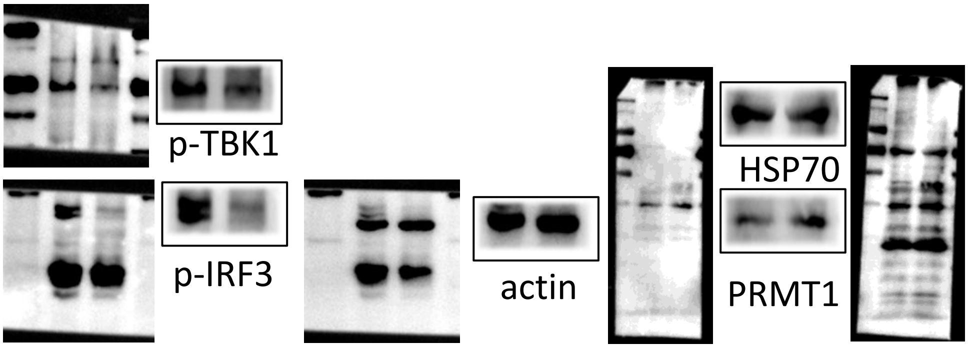

Fig4.K

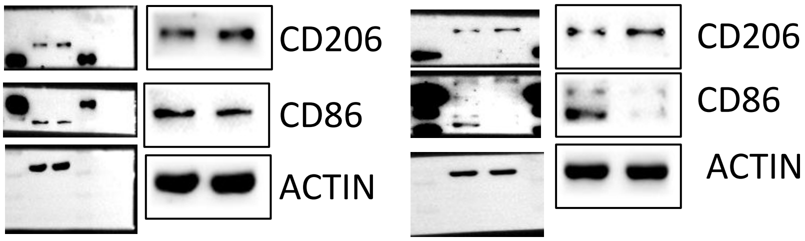

Fig5.A

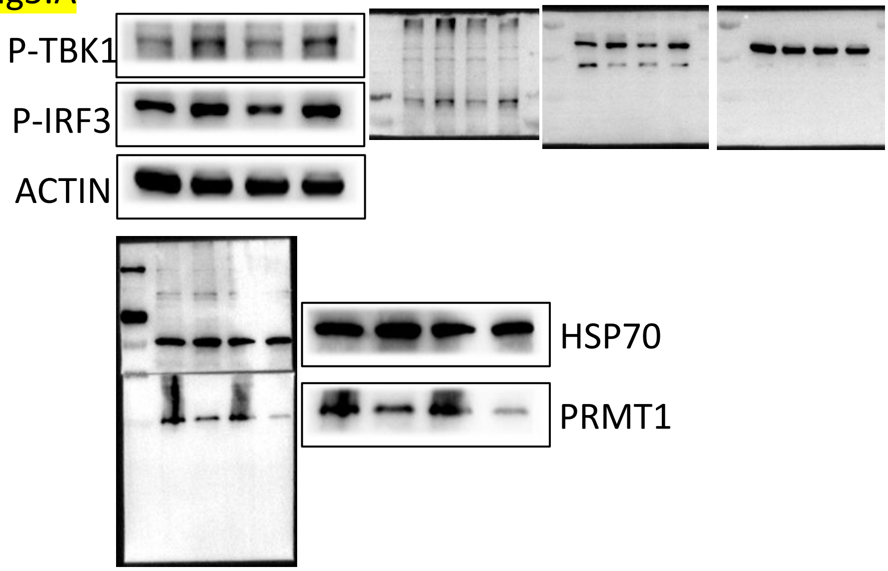

Fig5.I

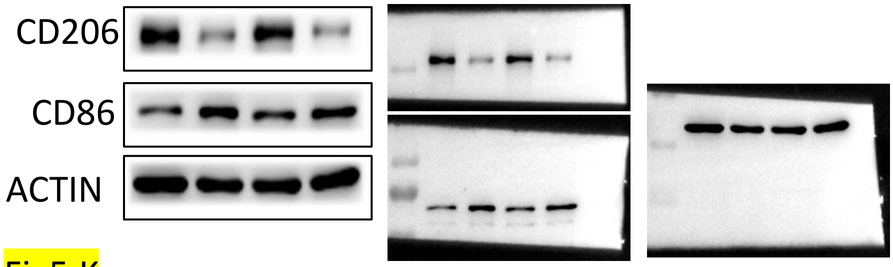

Fig5.K

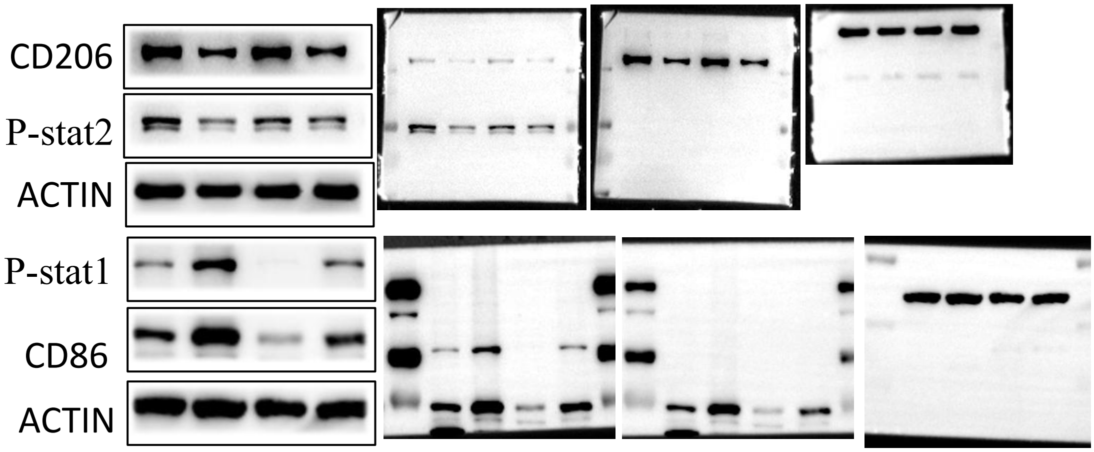

sup-Fig1.D

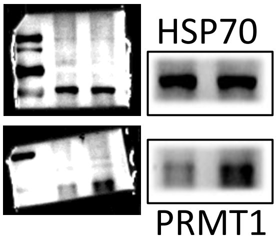

sup-Fig1.I

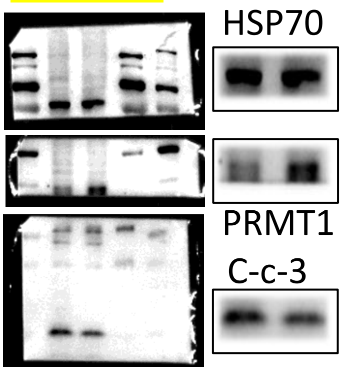

Sup.Fig2.A

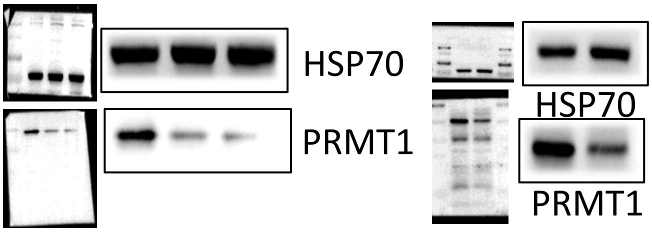

Sup.Fig2.J

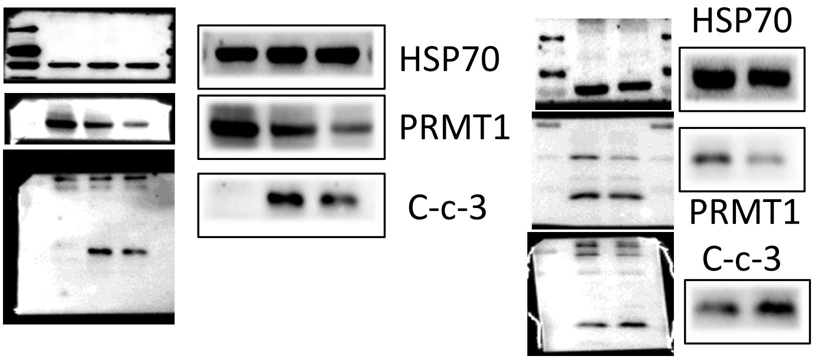

sup-Fig2.K

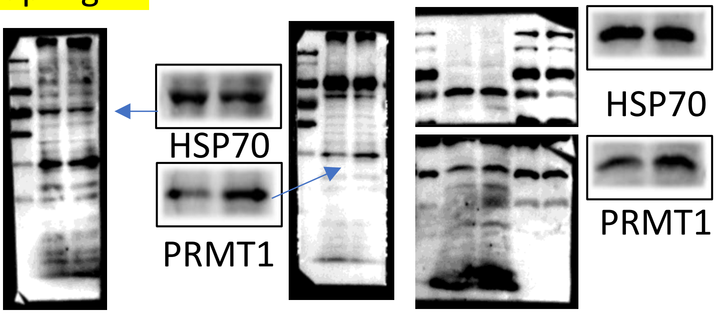

sup-Fig2.T

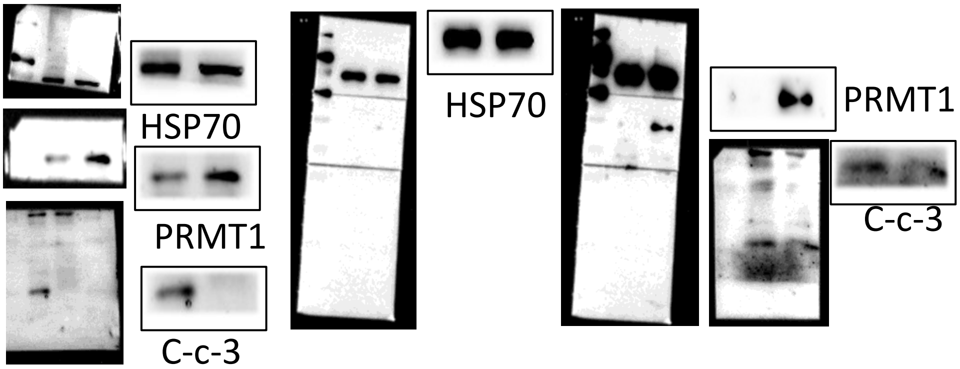

Sup-Fig2.U

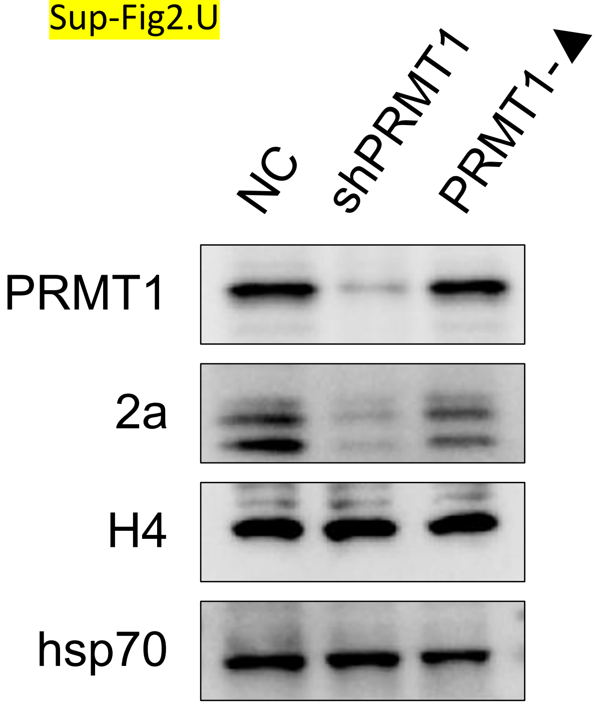

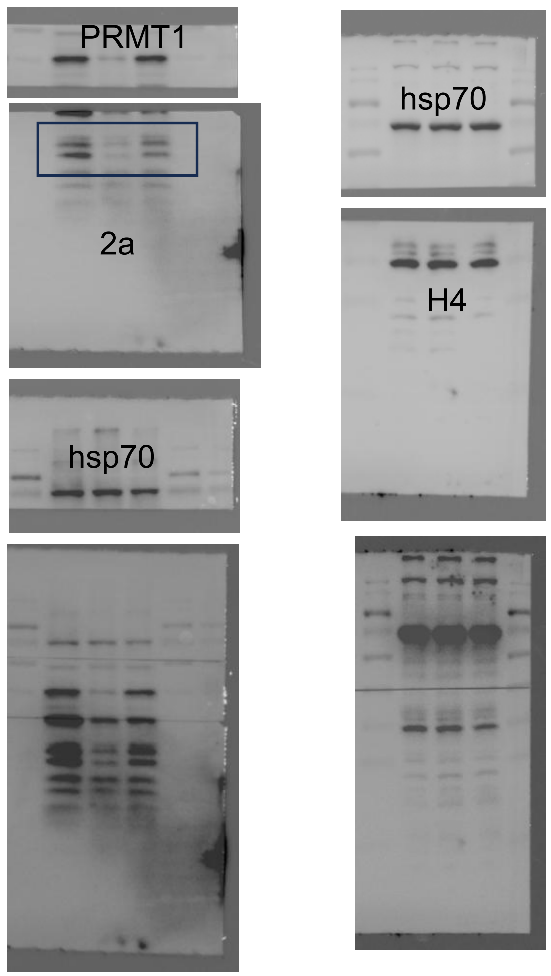

sup-Fig3.A

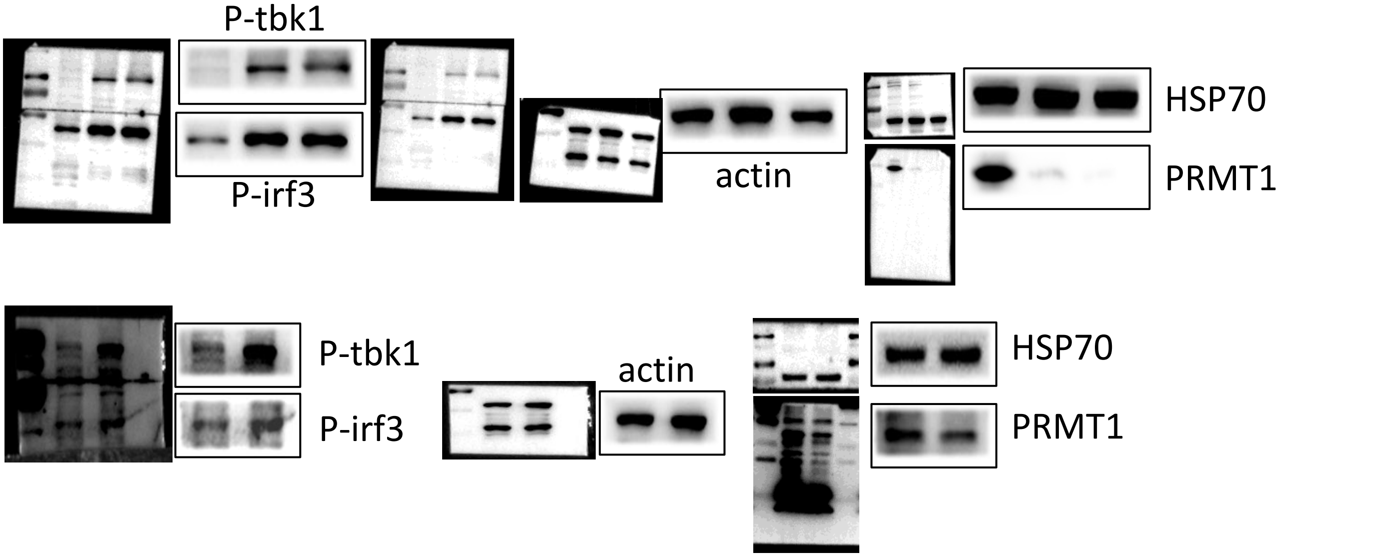

sup-Fig3.F

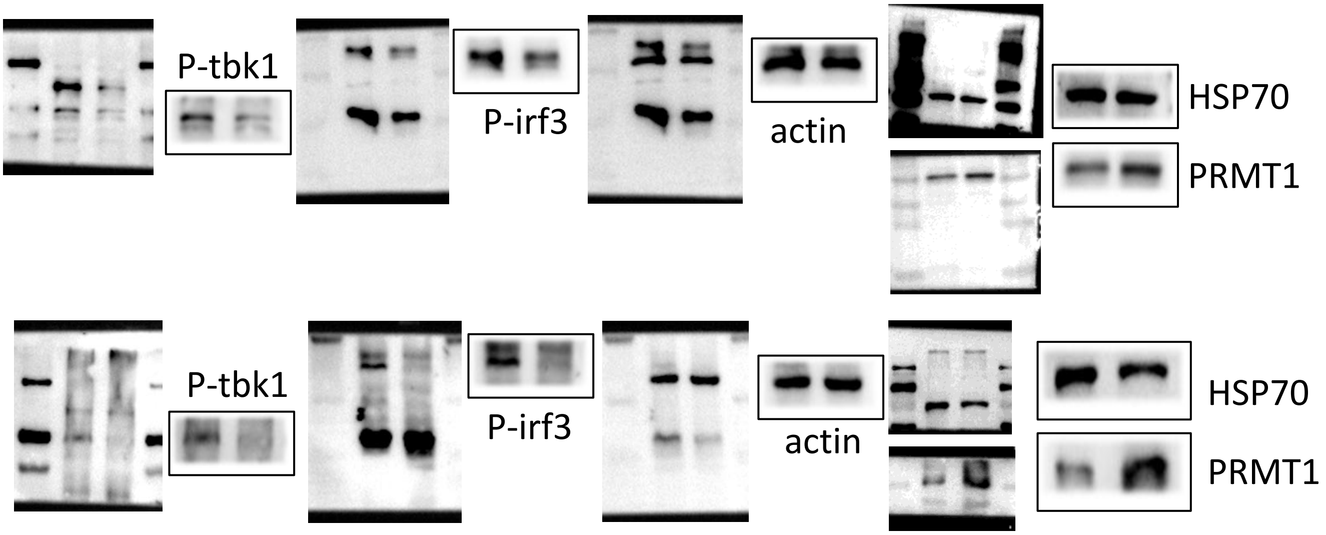

SUP-Fig6.A

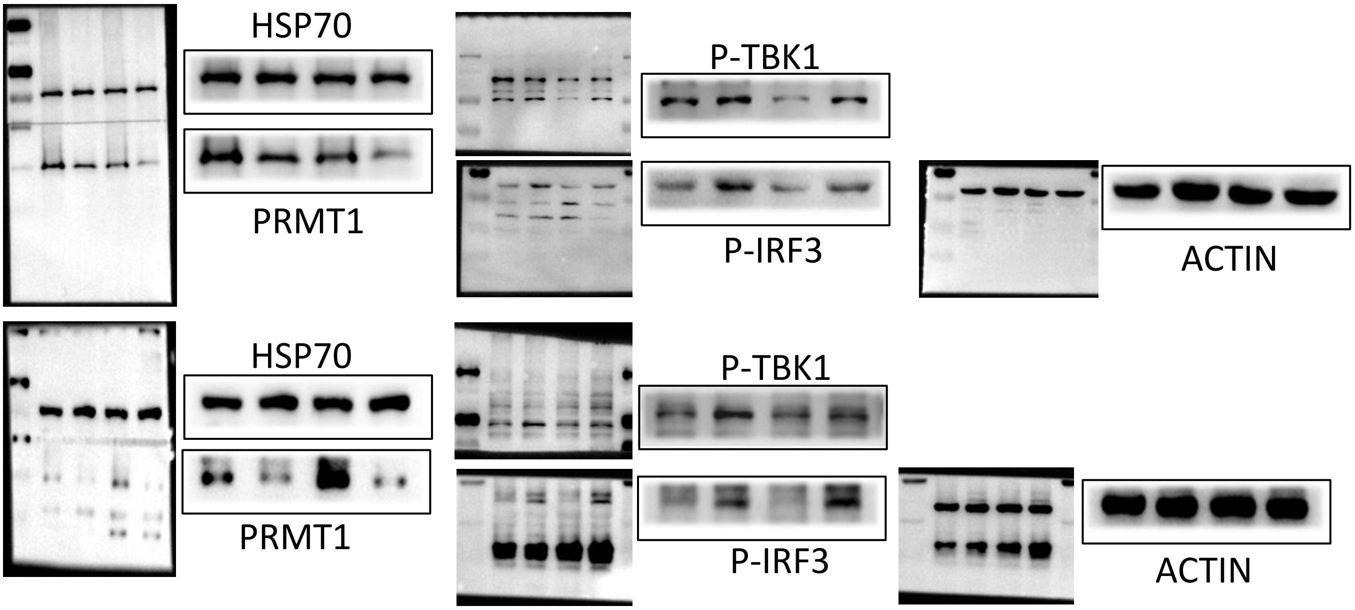

Sup-Fig7.C

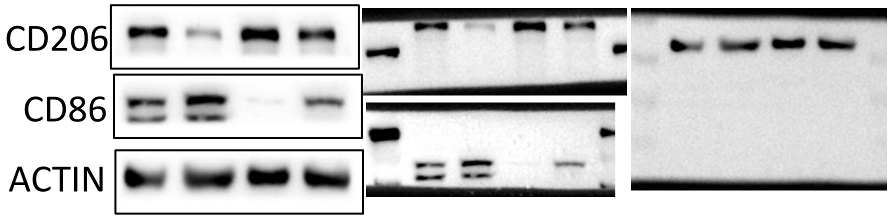

Sup-Fig7.E

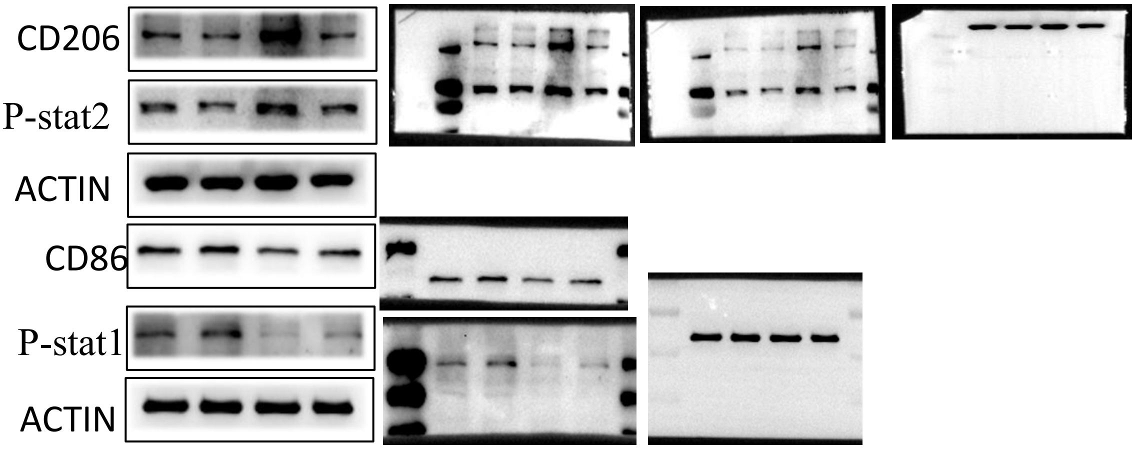

Supplement: Supplementary file 10 — Original western blots of this manuscript. [file 41419_2025_7960_MOESM10_ESM.pdf]
