# Supplementary material for: Tumor-derived PRMT1 suppresses macrophage antitumor activity by inhibiting cGAS/STING signaling in gastric cancer cells
Source: Cell Death Dis. 2025 Aug 26;16(1):649. doi: 10.1038/s41419-025-07960-y (PMC12381180; doi:10.1038/s41419-025-07960-y)
Supplement: Supplementary file 11 — Supplementary figure legend [file 41419_2025_7960_MOESM11_ESM.docx]

**Fig. S1: PRMT1 overexpression was linked to poor prognosis in GC patients and promoted the progression of HGC27 cells.**

(A) The volcano chart of RNA-seq in 4 pairs of cancer and paracancer tissue in GC patients. (blue: 365 down-regulating genes; red: 2259 up-regulated genes). (B - C) Kaplan-Meier curves of FP and PPS in GC patients, stratiﬁed by PRMT1 expression (Log-rank test, P = 0.0063/0.054) (https://kmplot.com/analysis/index.php?p=service&cancer=gastric). (E - F) PRMT1 expression levels of HGC27 after lentivirus interference detected by Western blots (E) and qRT-PCR (F). (F - G) Flow cytometry analysis of the apoptotic cells after overexpression of PRMT1 in HGC27 cells using the Annexin V-APC/PI staining assay (F). Positive Annexin V cells were displayed as histogram (G). (H) The proliferation rates of HGC27 cells upon overexpression of PRMT1 determined by CCK8 assay. (I) Western blots detected protein levels of PRMT1 and cleaved caspase-3 in HGC27 after up-expressing PRMT1. **P* < 0.05，***P* < 0.01，****P* < 0.001，*****P* < 0.0001.

**Fig. S2: Cancer promoting functions of PRMT1 in GC.**

(A - C) PRMT1 expression levels of AGS and MFC after lentivirus interference detected by Western blots (A) and qRT-PCR (B - C). (D - E) Flow cytometry analysis of the apoptotic cells after depletion of PRMT1 in AGS cells using the Annexin V-APC/PI staining assay (D). Positive Annexin V cells were displayed as a histogram (E). (F - G) Flow cytometry analysis of the apoptotic cells after depletion of PRMT1 in MFC cells using the Annexin V-APC/PI staining assay (F). Positive Annexin V cells were displayed as a histogram (G). (H - I) The proliferation rates of AGS (H) and MFC (I) upon depletion of PRMT1 were determined by CCK8 assay. (J) Western blots detected protein levels of PRMT1 and cleaved caspase-3 in AGS (left) and MFC (right) after down-expressing PRMT1. (K - M) PRMT1 expression levels of AGS and MFC after PRMT1 upregulated were detected by Western blots (K) and qRT-PCR (L - M). (N - O) Flow cytometry analysis of the apoptotic cells after overexpression of PRMT1 in AGS cells using the Annexin V-APC/PI staining assay (N). Positive Annexin V cells were displayed as a histogram (O). (P) The proliferation rates of AGS cells upon overexpression of PRMT1 were determined by CCK8 assay. (Q - R) Flow cytometry analysis of the apoptotic cells after overexpression of PRMT1 in MFC cells using the Annexin V-APC/PI staining assay (Q). Positive Annexin V cells were displayed as a histogram (R). (S) The proliferation rates of MFC upon overexpression of PRMT1 were determined by CCK8 assay. (T) Western blots detected protein levels of PRMT1 and cleaved caspase-3 in AGS (left) and MFC (right) after up-expressing PRMT1. (U) Western blots detected protein levels of PRMT1, H4R3me2a, and H4 in MFC after down-expressing PRMT1 or an enzyme activity mutant. (V - W) Flow cytometry analysis of the apoptotic cells after down-expression of PRMT1 or enzyme activity mutant in MFC cells using the Annexin V-APC/PI staining assay (V). Positive Annexin V cells were displayed as a histogram (W). (X) The proliferation rates of MFC upon down-expression of PRMT1 or an enzyme activity mutant were determined by CCK8 assay. *P < 0.05，**P < 0.01，***P < 0.001，****P < 0.0001.

**Fig. S3: PRMT1 knockdown triggered an antitumor response via the activation of cGAS/STING signaling.**

(A) Protein levels of PRMT1, p-TBK1, and p-IRF3 in AGS and MFC cells after PRMT1 was downregulated were detected by Western blot. (B, D) The mRNA expression of CGAS, STING1, IFNB1, TBK1， and IRF3 in AGS and MFC cells after PRMT1 knockdown. (C, E) The expression levels of IFN-β in the secretion of AGS and MFC cells ELISA detected after PRMT1 knockdown. (F) Protein levels of PRMT1, p-TBK1, and p-IRF3 in AGS and MFC cells after PRMT1 up-regulated were detected by Western blot. (G, I) The mRNA expression of CGAS, STING1, IFNB1, TBK1, and IRF3 in AGS and MFC cells after PRMT1 overexpressed. (H, J) The expression levels of IFN-β in the secretion of AGS and MFC cells ELISA detected after PRMT1 overexpressed. (M) Representative images of HGC27 cells using PicoGreen (green) and DAPI (blue). Magnification: 200 ×. (K, N) For immunofluorescent staining, AGS (K) or MFC (N) were stained with anti-γH2AX antibody (red) and counter-stained with DAPI (blue) after PRMT1 was downregulated. Magnification: 200 ×. (L) Representative images of AGS cells using anti-dsDNA (L) antibody (red) and counter-stained with DAPI (blue) (left). Relative fluorescence intensity of dsDNA to DAPI was displayed as a histogram (right). Magnification: 200 ×. (M, O) For immunofluorescent staining, AGS (M) or MFC (O) were stained with PicoGreen (green) and DAPI (blue) after PRMT1 was downregulated. Magnification: 200 ×. (P - Q) Representative images of comet assay of AGS or MFC cells after PRMT1 was downregulated. Magnification: 100 ×. *P < 0.05，**P < 0.01，***P < 0.001，****P < 0.0001.

**Fig. S4: PRMT1 amplifies cytosolic DNA accumulation and promotes innate immune activation in GC.**

(A - B) Histogram of KEGG pathway (A) and Reactome annotations analysis (B) from RNA-seq in HGC27 cells following knocking down PRMT1. (C) GSEA plots for cytosolic DNA-sensing pathway-related genes from RNA-seq in 4 pairs of cancer and paracancer tissue in GC patients. (D) Kaplan-Meier curves of overall survival in 53 patients with GC in the tissue chip, stratiﬁed by CD206^+^ cells per area (Log-rank test, P = 0.9444). (E - F) The correlation of PRMT1 expression and immune infiltration (macrophage(E), B cells, CD4^+^ T cells, CD8^+^ T cells, Treg^+^ T cells, and myeloid dendritic cells) in GC patients in TCGA by TIMER2. *P < 0.05，**P < 0.01，***P < 0.001，****P < 0.0001.

**Fig. S5:** **PRMT1 enzyme activity - induced antitumor properties leads to increased infiltration and polarization of M1-like TAMs in GC to improve antitumor ability.**

(A - B) BMDMs from the coculture system with MFC cells depleted or an enzyme activity mutant of PRMT1 and analyzed by flow cytometry with anti-CD86 and anti-CD206 antibodies (A). The ratio of MFI of CD86^+^ cells and CD206^+^ cells is shown (B). (C) Resected tumors formed in nude mice injected with the indicated cells from each group (n=3) were photographed. (D - E) The volumes and weights of resected tumors from each mouse were recorded. (F - G) Single cell suspension of resected tumors analyzed by flow cytometry with anti-CD86 and anti-CD206 antibodies (F). The ratio of MFI of CD86^+^ cells and CD206^+^ cells is shown (G). ^**^*P* < 0.01，^***^*P* < 0.001，^****^*P* < 0.0001.

**Fig. S6:** **PRMT1 knockdown-induced antitumor properties were abolished after inhibition of cGAS/STING signaling.**

(A) Expression levels of p-TBK1, and p-IRF3 after PRMT1 or (and) STING knockdown detected by Western blot in AGS and MFC cells. (B - C) Expression levels of *CGAS, STING, IFN-α, TBK1, and IRF3* after PRMT1 or (and) STING knockdown detected by qRT-PCR in AGS and MFC cells. (D - G) Flow cytometry analysis of the apoptotic cells after depletion of PRMT1 or (and) STING, in AGS (D) and MFC (F) cells using the Annexin V-APC/PI staining assay. Positive Annexin V cells were displayed as a histogram (E, G). (H - I) The proliferation rates of AGS (H) and MFC (I) upon depletion of PRMT1 or (and) STING determined by CCK8 assay. (J - K) The expression levels of IFN-β on secretion of AGS (J) and MFC (K) after depletion of PRMT1 or (and) STING detected by ELISA. ^*^*P* < 0.05，^**^*P* < 0.01，^***^*P* < 0.001，^****^*P* < 0.0001.

**Fig. S7：** **PRMT1 knockdown-induced antitumor properties and polarization of M1-like macrophages through STAT signaling were abolished after inhibition of cGAS/STING signaling.**

(A) Tumor macroscopical images at the end of the experiments in four groups with or without H151 treatment. (E) Representative microphotographs of IHC staining of PRMT1, cleaved caspase-3, p-TBK1, p-IRF3, CD86^+^, and CD206^+^ cells in the upper 4 groups. (A - B) BMDMs from the coculture system with MFC cells depleted or not depleted of PRMT1 or (and) STING and analyzed by flow cytometry with anti-CD86 and anti-CD206 antibodies (A). The ratio of MFI of CD86^+^ cells and CD206^+^ cells is shown (B). (C) Western blots detected protein levels of CD86 and CD206 in BMDMs from the coculture system with MFC cells depleted of PRMT1 or (and) STING. (D) mRNA levels of M1 and M2 macrophage markers in BMDMs from the coculture system with MFC cells after PRMT1 or (and) STING knockdown detected by qRT-PCR. (E) Western blots detected protein levels of CD86, CD206, p-STAT1, and p-STAT2 in BMDMs from the coculture system with MFC cells depleted of PRMT1 or (and) STING. (F - G) Flow cytometry analysis of the apoptotic MFC cells after depletion of PRMT1 or (and) STING in MFC cells from the coculture system with BMDMs using the Annexin V-APC/PI staining assay (F). Positive Annexin V cells were displayed as a histogram (G). (H - I) Single-cell suspensions from orthotopic tumors of MFC cells, depleted or not depleted of PRMT1 or (and) treated with H151, and analyzed by flow cytometry with anti-CD86 and anti-CD206 antibodies (H). The ratio of MFI of CD86^+^ cells and CD206^+^ cells is shown (I). *P < 0.05，**P < 0.01，***P < 0.001，****P < 0.0001.

**Fig. S8：** **PRMT1 inhibitor MS023 and macrophage-depleting agent clodronate liposomes on mice affects xenograft gastric cancer growth in vivo.**

(A) Resected tumors formed in nude mice injected with the indicated cells from each group treated with or without PRMT1 inhibitor MS023 and macrophage-depleting agent clodronate liposomes (n=3) were photographed. (B - C) The volumes and weights of resected tumors from each mouse were recorded. (D - E) Flow cytometry analysis of Single-cell suspensions from orthotopic tumors with anti-CD11b and anti-F4/80 antibodies (D). The ratio of MFI of macrophages is shown (E). ^*^*P* < 0.05，^**^*P* < 0.01，^***^*P* < 0.001，^****^*P* < 0.0001.
